# Supplementary material for: Deep learning-based aberration compensation improves contrast and resolution in fluorescence microscopy
Source: bioRxiv. 2023 Oct 24:2023.10.15.562439. Preprint. [Version 2] doi: 10.1101/2023.10.15.562439 (PMC10659418; doi:10.1101/2023.10.15.562439)
Supplement: Supplement 1 [file media-1.pdf]

Supplementary Video Captions

Supplementary Video 1, Lateral views of synthetic phantom data restored by DeAbe vs. other methods. Phantoms consisting of randomly oriented and positioned dots, lines, spheres, spherical shells, and circles (ground truth, GT) were blurred to simulate microscopy data (Raw) and restored using blind deconvolution (Blind decon), Richardson-Lucy deconvolution with diffraction-limited PSF (RL Decon 1), Richardson-Lucy deconvolution with aberrated PSF (RL Decon 2), or our de-aberrating network (DeAbe). Lateral views though the volume are shown. Twenty iterations were used for RL deconvolution and 10 for blind deconvolution. See also **Fig. 1.**

Supplementary Video 2, Axial views of synthetic phantom data restored by DeAbe vs. other methods. As in **Supplementary Video 1**, but showing axial views through the volume.

Supplementary Video 3, DeAbe restores images of *C. elegans* embryos expressing nuclear marker. Images were acquired with single view light sheet microscopy (iSPIM, 1.1NA). Left: raw data, middle: same data after 10 iterations of Richardson-Lucy deconvolution (RL Decon), right: restoration after DeAbe. Lateral views through the image volume are shown. See also **Fig. 2a**.

Supplementary Video 4, DeAbe restores images of adult *C. elegans* expressing NeuroPAL;GCaMP6s. Images were acquired with spinning disk confocal microscopy. Left: raw data, right: restoration after DeAbe. Lateral views of individual color channels (1st – 3rd row) and combined channels (4th row) through the image volume are shown. Note contrast has been increased to better visualize dim nuclei, this results in a large background in the red channel in the last few planes. See also **Supplementary Fig. 7**.

Supplementary Video 5, DeAbe restores images of NK-92 cells fixed and stained with Alexa Fluor 555 wheat germ agglutinin. Images were acquired with instant SIM. Volumetric maximum intensity projections (MIP), lateral views, and axial views through the image volume are shown in sequence. Left: raw data, middle: same data after restoration with DeAbe, right: same data after restoration with DeAbe+ (DeAbe followed by 20 iterations Richardson-Lucy deconvolution). See also **Fig. 2b-d**.

Supplementary Video 6, DeAbe restores lateral views of live cardiac tissue expressing GFP-Tomm20. Images were acquired with two photon microscopy. Left: raw data, middle: after 20 iterations of Richardson-Lucy deconvolution (RL Decon), right: restoration after DeAbe. Lateral views through the volume are shown. See also **Fig. 2e-f**.

Supplementary Video 7, DeAbe restores axial views of fixed tissue expressing tdTomato membrane marker. Images were acquired with two photon microscopy. Left: raw data, right: restoration after DeAbe.

Supplementary Video 8, DeAbe ameliorates image degradation in mm-scale cleared mouse tissue embryo. Fixed and CLARITY-cleared E11.5-day mouse embryo was immunostained for blood vessels (CD31, magenta) and neurons (TuJ1, cyan). Rendering compares raw and DeAbe+ (attenuation compensated, DeAbe, and deconvolved) data. See also **Fig. 3a-d**.

Supplementary Video 9, DeAbe enhances quantification of vessel orientation and alignment in mm-scale cleared mouse tissue embryo. Orientations (theta and phi) and 3D directional variance (DV) analysis on the blood vessel channel of the mouse embryo data in **Supplementary Video 8**. Rendering

compares raw and DeAbe+ (attenuation compensated, DeAbe, and deconvolved) results. See also **Fig. 3e-g**.

Supplementary Video 10, Multi-step deep learning restores images of *C. elegans* embryos expressing nuclear (magenta) and membrane (green) markers. Images were acquired with single view light sheet microscopy (iSPIM, 1.1NA) and restored using a three-step deep learning pipeline. Maximum intensity projections (MIP, left column, shown only for nuclear channel) and single lateral plane 17.7μm into the volume (right column, both nuclei and membranes) are shown for raw (top row) and restored (bottom row) data. See also **Fig. 4a, b**.

Supplementary Video 11, Multi-step deep learning improves image quality and cell segmentation for *C. elegans* embryos. The 80th time point volume (~320 min post fertilization) extracted from the time series data in **Supplementary Video 10**. Top row shows raw image (top left) and restored image (top right) using three-step deep learning pipeline, with nuclei in magenta and membrane in green. Bottom row shows the automated cell segmentation based on the raw image (bottom left) and the automated cell segmentation followed by manual editing based on restored image (bottom right). 319 cells are automatically segmented in the raw data, but 421 cells are segmented based on manual editing of the multi-step deep learning result. Lateral views through the image volume are shown. See also **Fig. 4c**.

Supplementary Video 12, Multi-step deep learning restores images of *C. elegans* embryos expressing ttx-3B-GFP, marking neurons and gut cells. Raw images (left) were acquired with single view light sheet microscopy (iSPIM, 0.8NA) and restored using three-step deep learning pipeline. Maximum intensity projections through time are shown; selected time points also show volumetric projections. See also **Fig. 4i**.

Supplementary Video 13, Multi-step deep learning restores images of *C. elegans* embryos expressing nuclear marker. Images were acquired with single view light sheet microscopy (iSPIM, 1.1NA) and restored using three-step deep learning pipeline. From left to right were shown the raw images, Step 1 DeAbe, Step 2 Decon, and Step 3 Expan results. Lateral views through the volume are shown.

Supplementary Video 14, Multi-step deep learning restores time-lapse images of *C. elegans* embryos expressing nuclear marker. Images were acquired with single view light sheet microscopy (iSPIM, 1.1 NA) and restored using three-step deep learning pipeline. From left to right were shown the raw images, Step 1 DeAbe, Step 2 Decon, and Step 3 Expan results. Maximum intensity projections through time are shown.

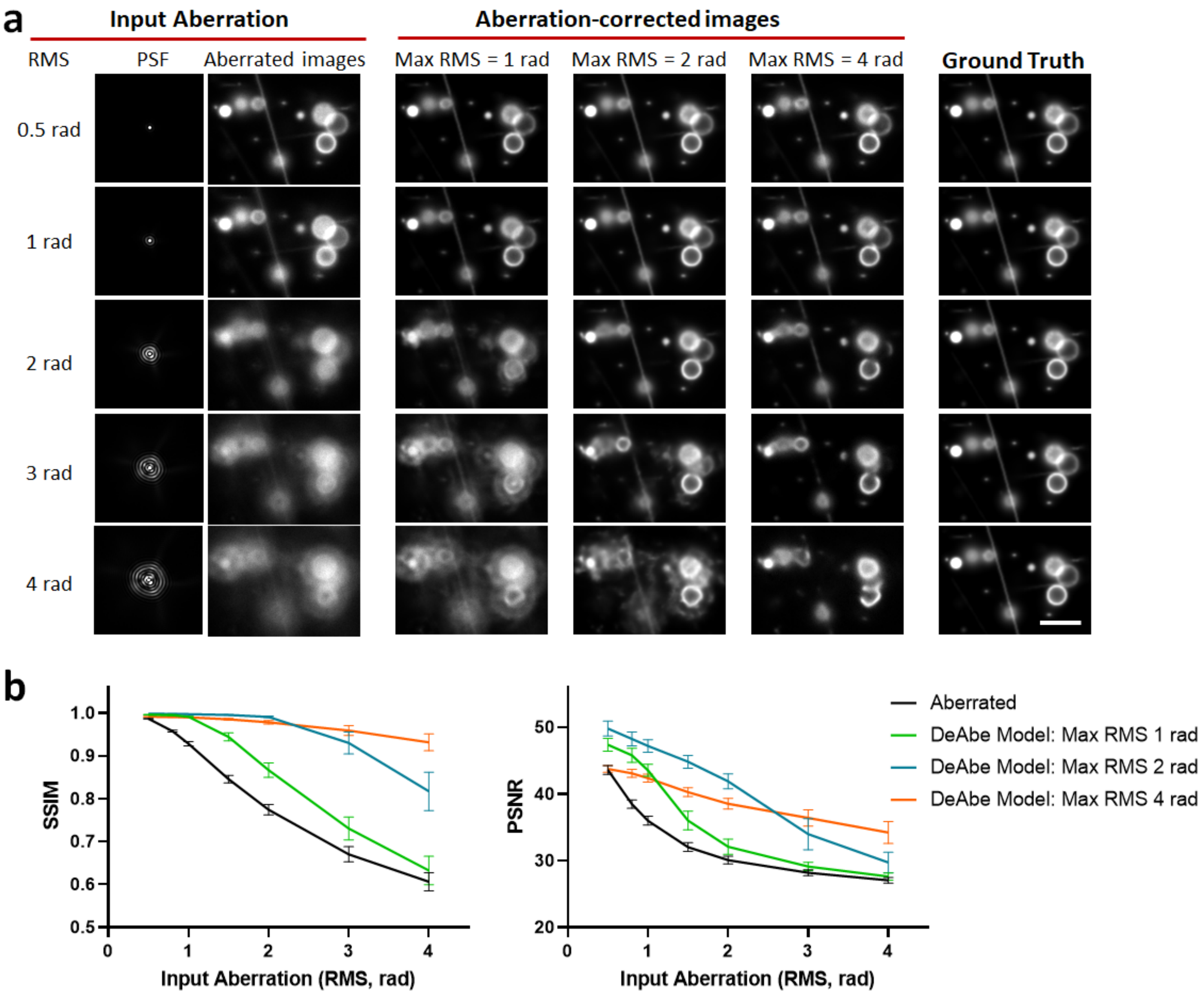

Supplementary Fig. 1, DeAbe performance depends on aberration magnitude in input images and training data. A) Example aberrated PSFs and associated aberrated input images (left); DeAbe model predictions yielding aberration corrected images (middle), shown for different training data consisting of mixed aberrations with indicated maximum root mean square (RMS) wavefront distortion (see also **Methods**); and ground truth synthetic objects (right). The example input aberration here is defocus, applied at an increasing aberration magnitude (0.5 rad – 4 rad RMS wavefront distortion). **b)** Quantification of **a)**. Structural similarity index (SSIM) and peak signal-to- noise ratio (PSNR) metrics are used to quantify the quality of DeAbe Net predictions under different training data regimes (green, blue, red curves at 1, 2, and 4 rad max RMS) vs. ground truth. All DeAbe predictions show improvement compared to input aberrated image (black), with training data containing larger magnitude aberrations better able to compensate for larger aberration magnitudes in the input images. For this work, we used the model corresponding to a maximum RMS wavefront distortion of two radians (blue curve). Means

and standard deviations are shown from 100 independent simulations, each with randomized object structures and randomized input aberrations. Scale bar: 5 μm.

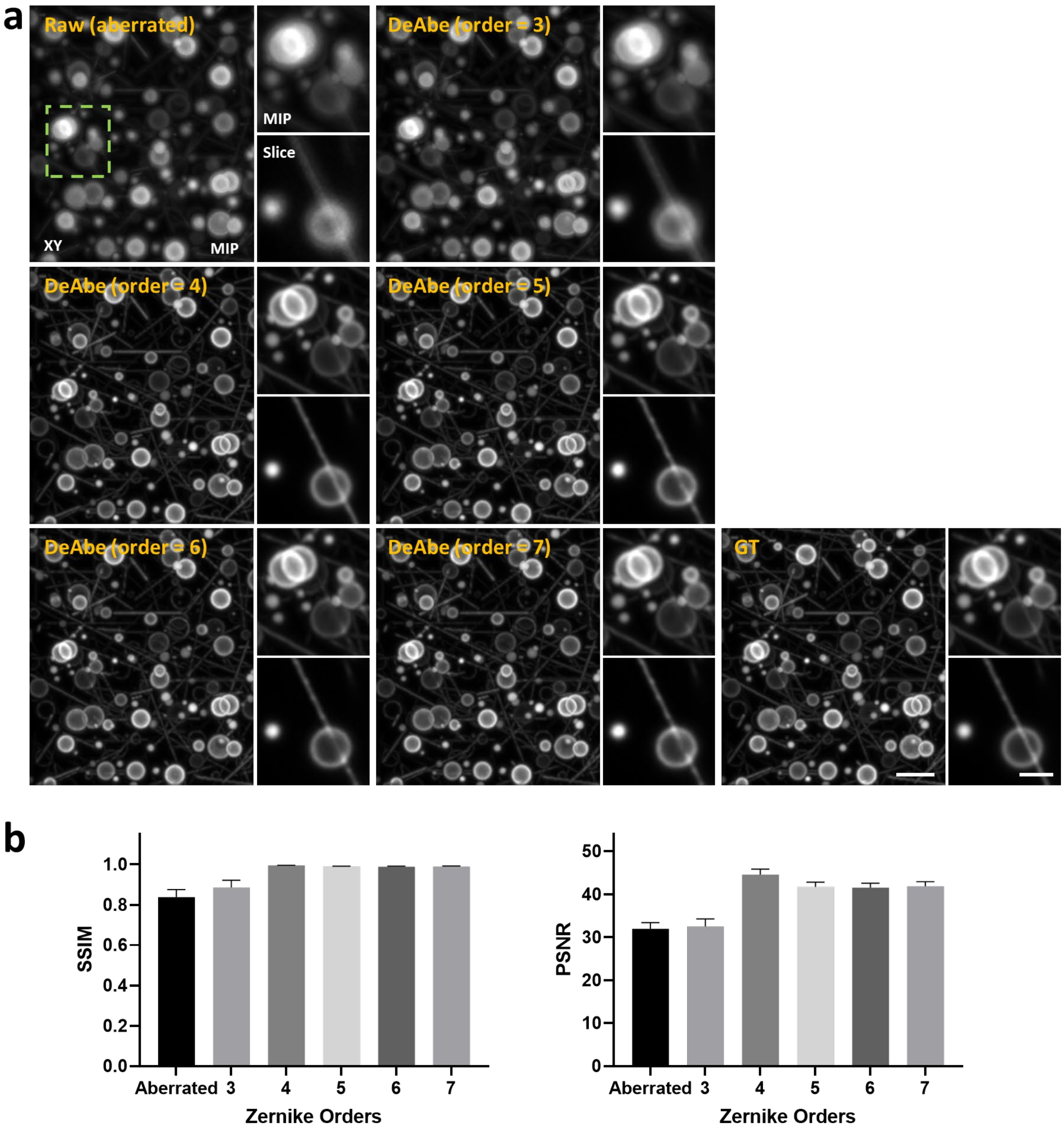

Supplementary Fig. 2, Dependence of DeAbe prediction on number of Zernike orders in training data. Synthetic aberrated phantom structures in **Fig. 1b** were input into DeAbe networks trained with progressively more Zernike basis aberrations. Visual **a)** and quantitative **b)** analysis indicate that performance plateaus after the 4th Zernike terms are added. Thus, in this work, we used four Zernike orders to generate training data. In **b)**, both SSIM (left) and PSNR (right) values are shown from 100 independent simulations. Scale bars: 5 μm and 2.5 μm (insets).

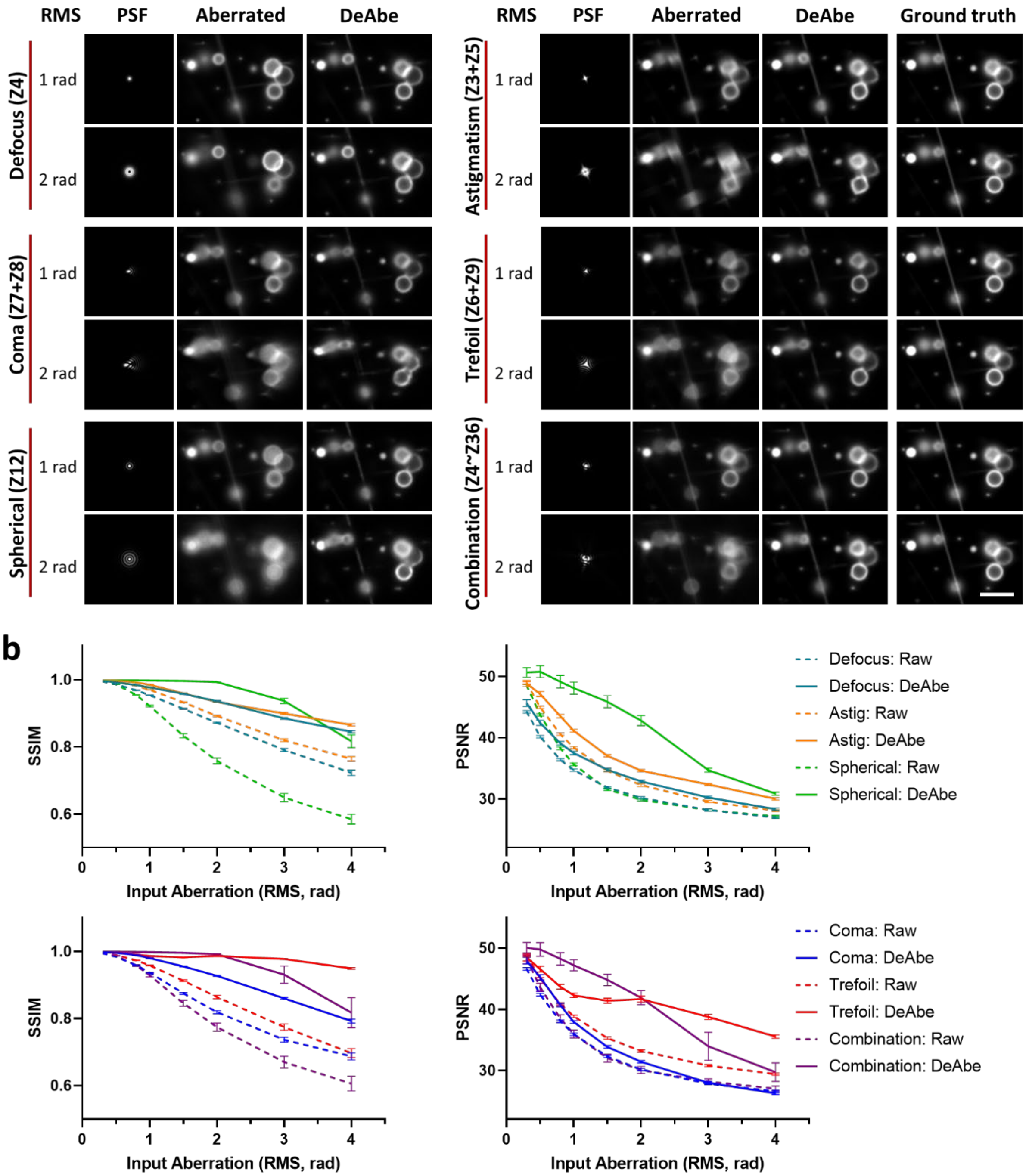

Supplementary Fig. 3, DeAbe network predictions on images contaminated with specific aberration modes. Synthetic phantoms were aberrated with defocus, coma, spherical, astigmatism, trefoil, and mixed aberrations with indicated RMS wavefront distortion. A DeAbe model trained on data contaminated with mixed aberrations (up to fourth order Zernike basis terms, maximum RMS wavefront distortion two radians) was then used to compensate for aberrations. **a)** Aberrated PSFs (left), aberrated images (middle) and DeAbe model predictions (right) are shown for each condition, as well as the ground truth reference (GT). Z in each vertical axis label refers to the Zernike order. **b)** Quantification

using SSIM and PSNR metrics (means and standard deviations from 100 independent simulations) support the visual improvement after application of the DeAbe model. Scale bar: 5 μm.

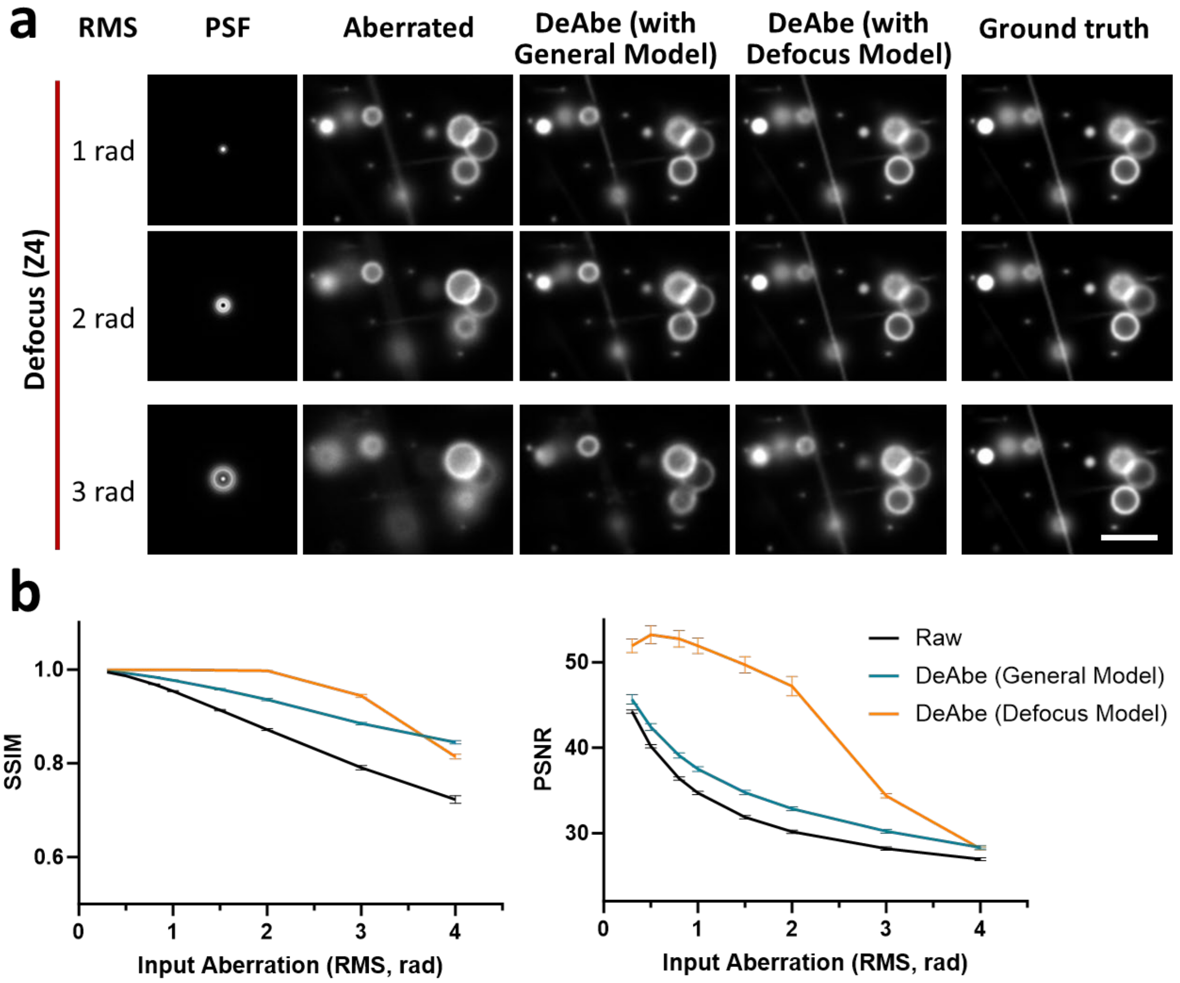

Supplementary Fig. 4, DeAbe network predictions on defocused images improve if using a dedicated network trained purely on defocused images. Synthetic phantoms were aberrated with defocus with indicated RMS wavefront distortion. A general DeAbe model trained on data contaminated with mixed aberrations (up to fourth order Zernike basis terms, maximum RMS wavefront distortion two radians), or a specific defocus DeAbe model trained on data contaminated with only defocus aberrations (maximum RMS wavefront distortion two radians), was then used to compensate for aberrations. **a)** From left to right: aberrated PSFs, aberrated images, predictions with a general DeAbe model, predictions with a specific defocus DeAbe model, and the ground truth reference (GT). **b)** Quantification using SSIM and PSNR metrics (means and standard deviations from 100 independent simulations) to compare the performance of the general DeAbe model vs. the specific defocus DeAbe model. Scale bar: 5 µm.

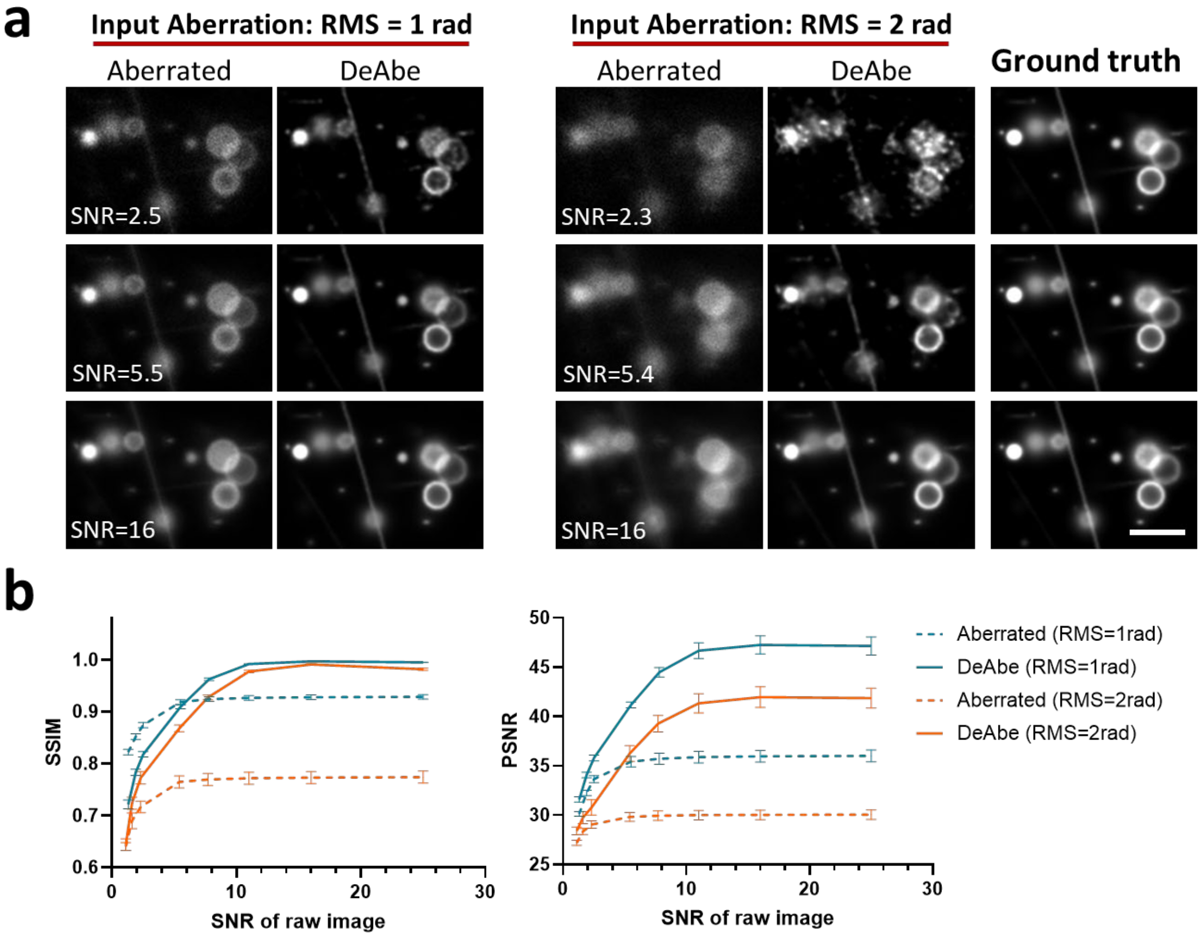

Supplementary Fig. 5, Effect of noise on DeAbe model prediction. a) Images of synthetic phantom structures with aberrations were additionally contaminated with noise to simulate different SNR levels and degraded with indicated RMS wavefront distortion. Ground truth structure (GT) is shown for comparison. **b)** SSIM and normalized PSNR analysis (means and standard deviations from 100 independent simulations) for data in **a),** confirming that performance of DeAbe network deteriorates in the presence of increasing noise and aberration. Scale bar: 5 μm.

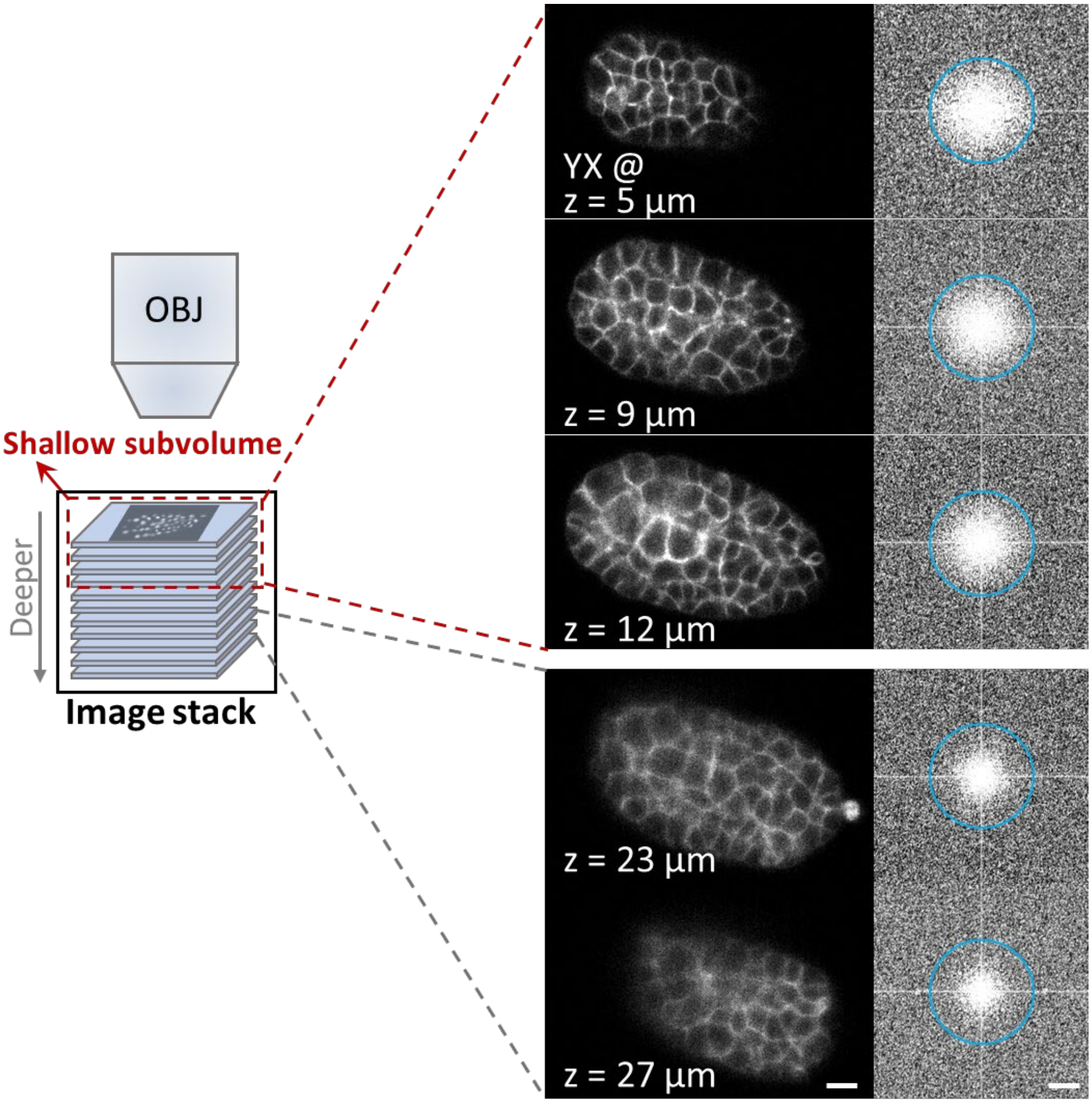

Supplementary Fig. 6, Shallow subvolume selection. Selected planes at different depths of an image stack from *C. elegans* embryos expressing membrane marker, with lateral views (left) and corresponding Fourier transforms (right) showing progressive degradation in image quality from the shallow side (closest to the detection objective) to the deeper side of the stack due to aberrations. The slices on shallow side (referred to as a “shallow subvolume”) are manually selected as ground truth and used to generate synthetically aberrated images. Blue circles in right panel indicate 1/0.6 μm^{-1} spatial frequency. Scale bars: 5 μm (left) and 1/2.5 μm^{-1}(right).

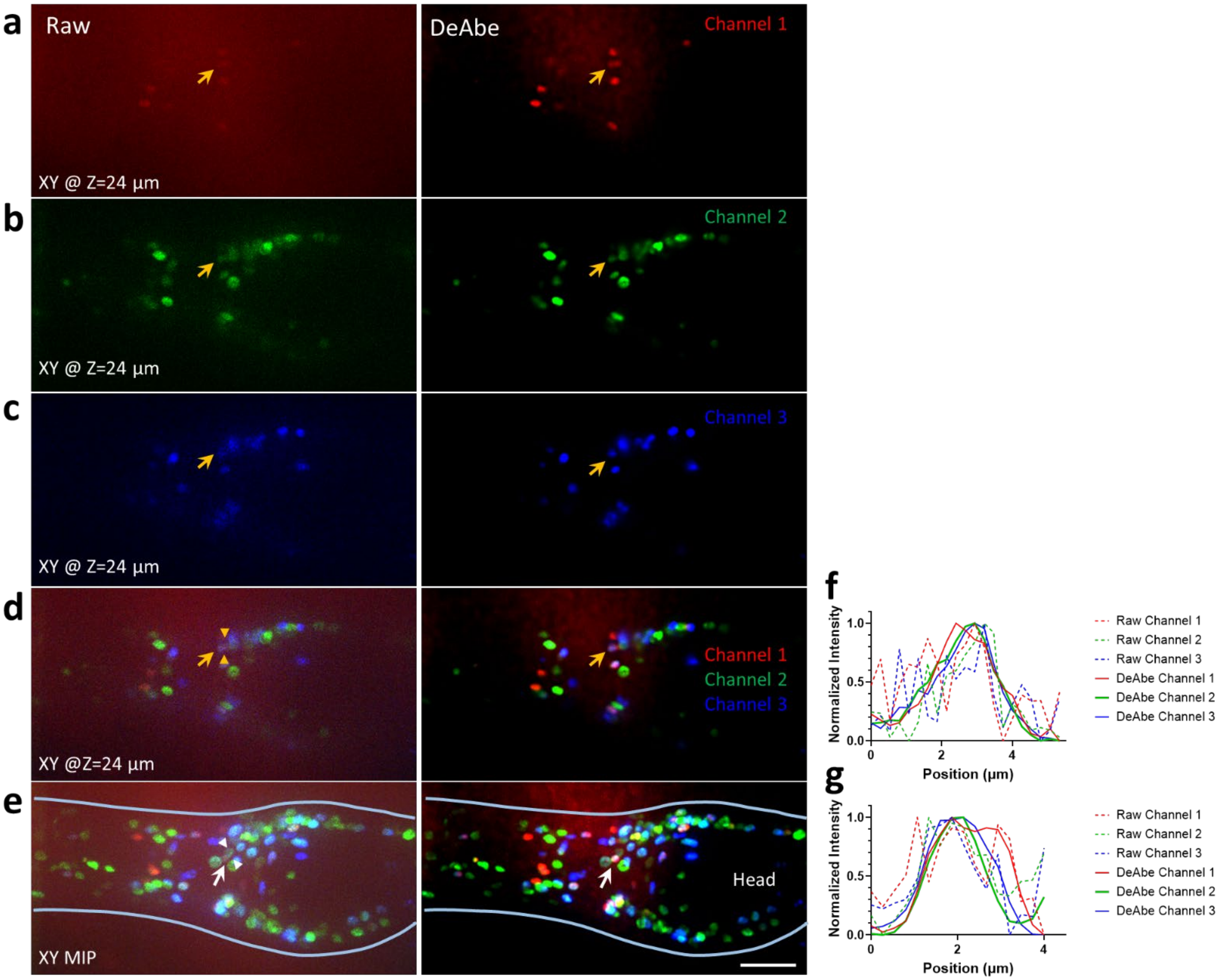

Supplementary Fig. 7, DeAbe compensates for aberrations in spinning-disk confocal microscopy. Adult *C. elegans* expressing NeuroPAL;GcaMP6s was imaged with spinning-disk confocal microscopy (**a-e)**, left) and our DeAbe model used to restore the data (**a-e)**, right). Individual planes 24 μm from the surface of the worm are shown in each color channel **a-c)**), in merged overlay **d)**, or a maximum intensity projection (MIP) over the whole volume **e)** are shown. In the MIP, the boundary of the worm has also been traced (blue lines) and the head region indicated for context. Orange (**a-d**) or white **e)** arrows highlight nuclei; double-headed arrowheads show line profiles (**f, g**) over the same nuclei comparing raw images (dashed lines) to DeAbe model prediction (solid lines). The network prediction improves signal-to-noise ratio and contrast in each channel compared to the raw data. Intensity profiles in (**f, g**) are each normalized to the maximum value in their profile. See also **Supplementary Video 4**. Scale bar: 20 μm.

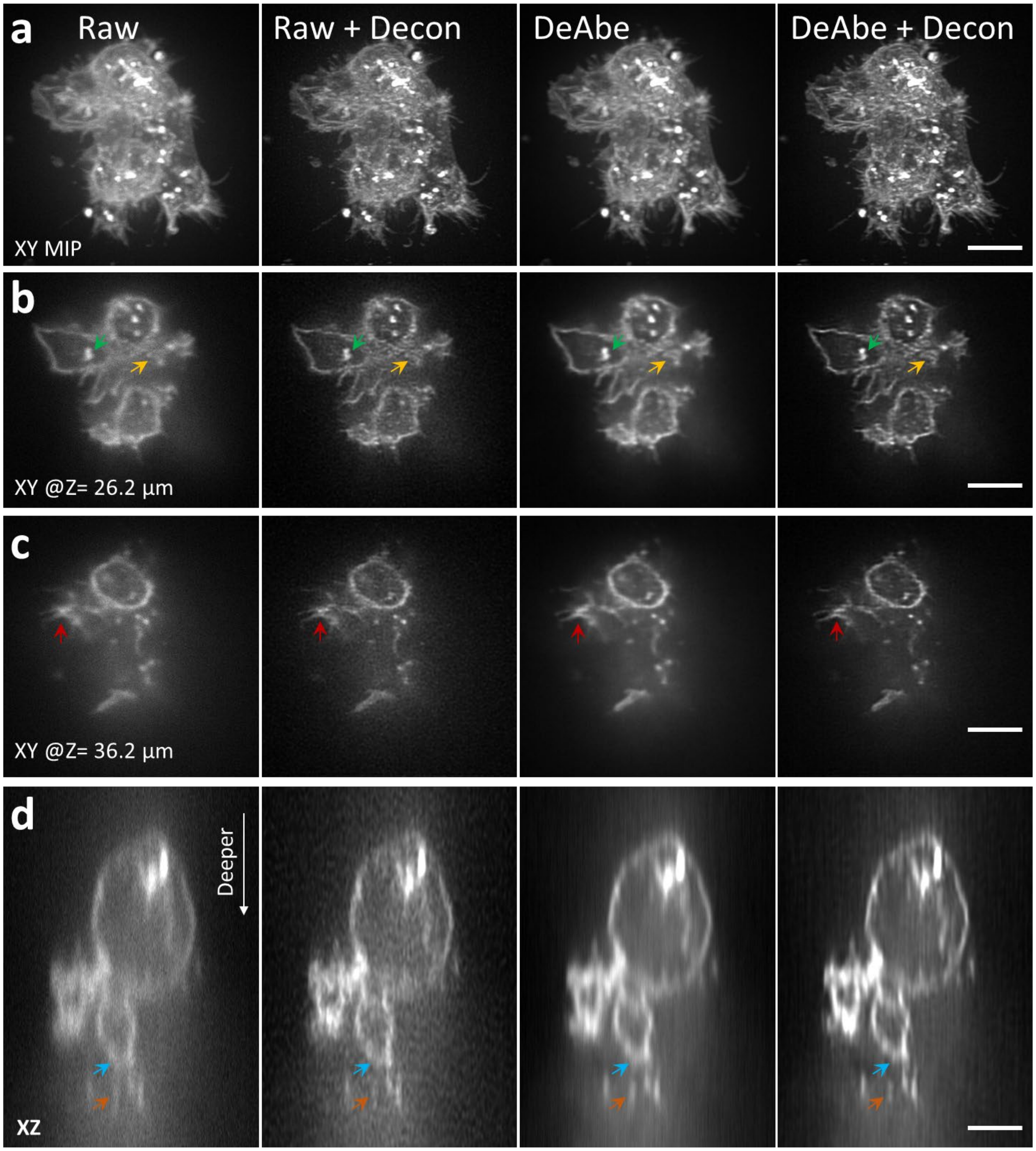

Supplementary Fig. 8, Progressive improvements in image quality in NK-92 cells imaged with iSIM. Cells were fixed and stained for wheat germ agglutinin as in **Fig. 2b-d**. Columns show (left to right) representative images of raw data, deconvolved data, DeAbe prediction on raw data, and DeAbe prediction followed by deconvolution (20 iterations Richardson-Lucy). The latter provides the clearest lateral (**a-c**) and axial (**d)** views, best highlighting fine features in the data (arrows). MIP: maximum intensity projection. Scale bars: 5 µm. See also **Supplementary Video 5**.

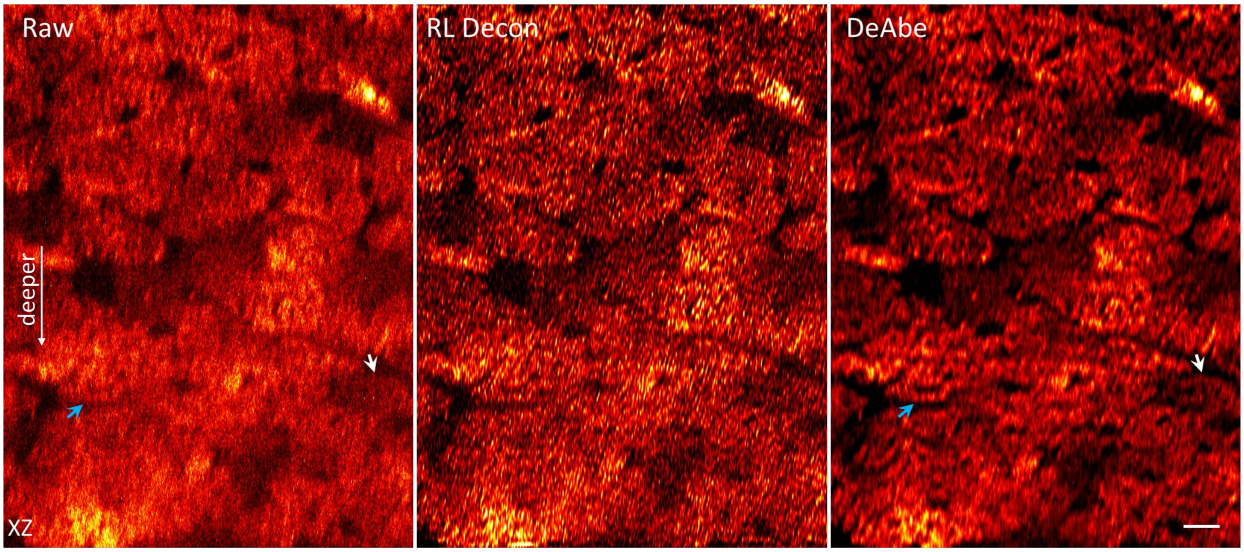

Supplementary Fig. 9, DeAbe compensates for aberrations in two photon microscopy. Live cardiac tissue containing cardiomyocytes expressing Tomm20-GFP imaged with two photon microscopy. Left: axial view of raw data with the direction of increasing depth indicated with white arrow. Middle: Richardson-Lucy deconvolution on the raw volume (20 iterations). Right: DeAbe model prediction. Arrows highlight space between cells or cell boundaries better delineated in prediction than raw data. See also **Supplementary Video 6**. Scale bar: 10 μm.

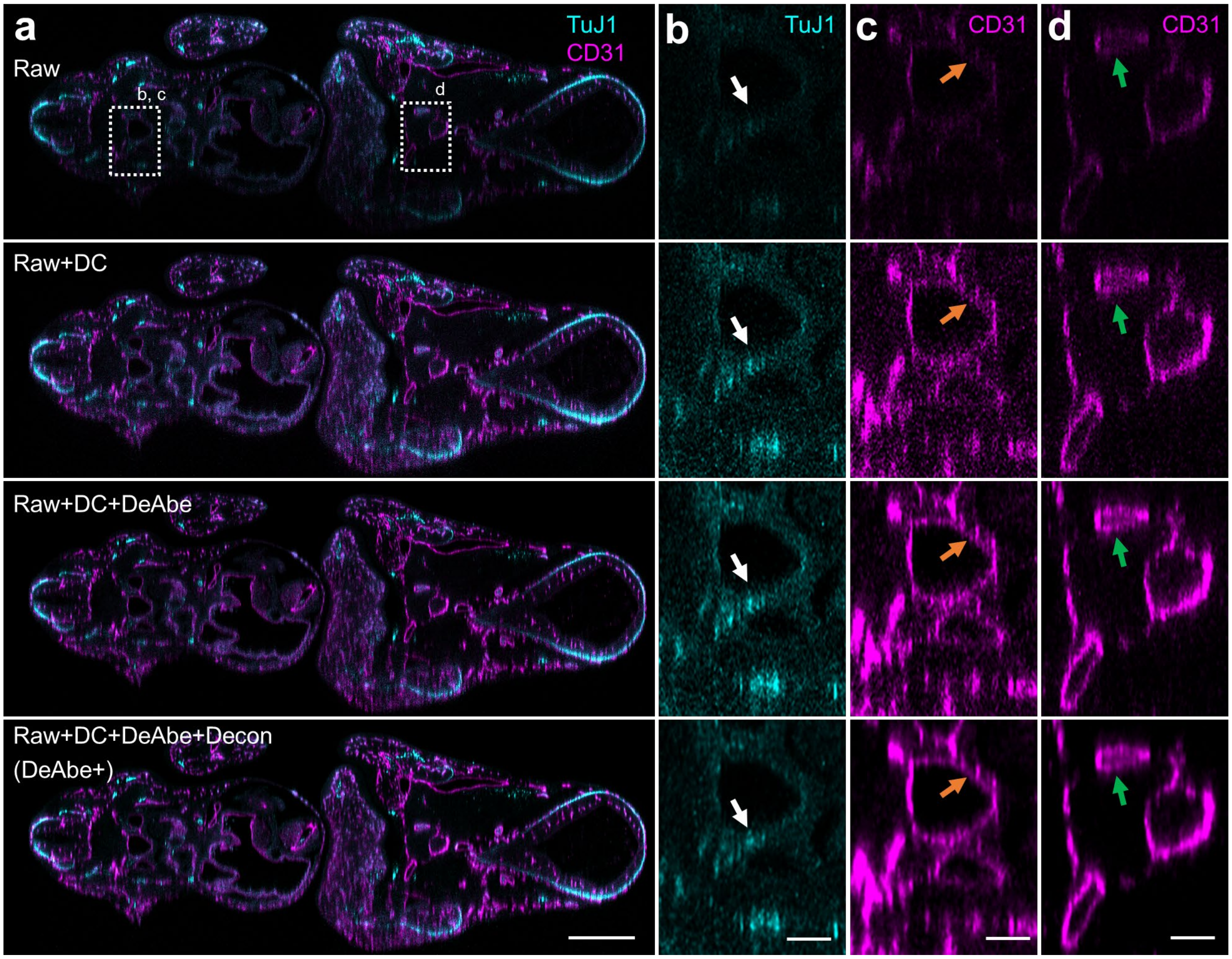

Supplementary Fig. 10, Progressive improvements in image quality in cleared mouse embryo images. **a)** Axial views of cleared mouse embryo corresponding to data in **Fig. 3b**, comparing (top to bottom) raw data; intensity decay compensated (DC, see also **Methods**); DC followed by application of DeAbe; and DC, DeAbe and deconvolution (‘DeAbe+’, 20 iterations Richardson-Lucy). Higher magnification views of dashed rectangular regions in **a)** are shown in TuJ1 channel (**b**) and CD31 channel (**c, d**). Arrows highlight boundaries that are progressively more clearly delineated after each restoration step. See also **Supplementary Video 8**. Scale bars: 500 μm **a)**; 100 μm **b, c, d)**.

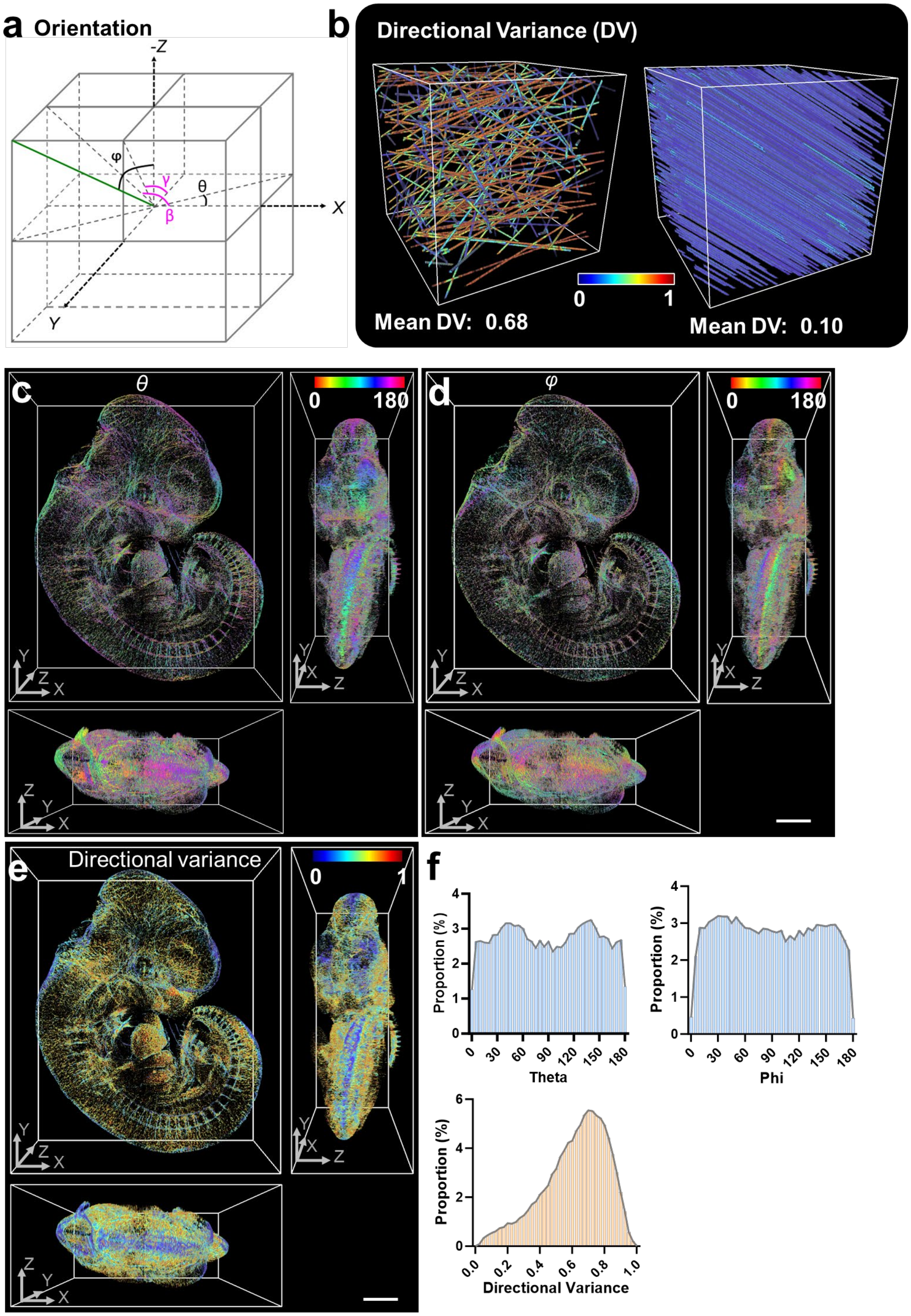

Supplementary Fig. 11, **Key variables in orientational vessel analysis. a)** Defining the azimuthal angle θ and polar angle φ variables for a certain orientation (the green line) with respect to Cartesian coordinate system, with two additional two azimuthal angles, β and γ, to assist the calculation of φ. **b)**

simulation of oriented lines with more (left) or less (right) mean directional variance (DV). More directional variance implies lines are more randomly oriented. **c-e)** Orientation analysis on blood vessel channel of DeAbe processed data of CLARITY-cleared E11.5-day mouse embryo, immunostained for neurons (TuJ1) and blood vessels (CD31). Perspective views of θ (**c**), φ (**d**) and DV (**e**) distributions in 3D are shown. **f**) Histograms of θ (top left), φ (top right), and DV (bottom). See also **Fig. 3e-g, Supplementary Video 9.** Scale bars: 500 μm.

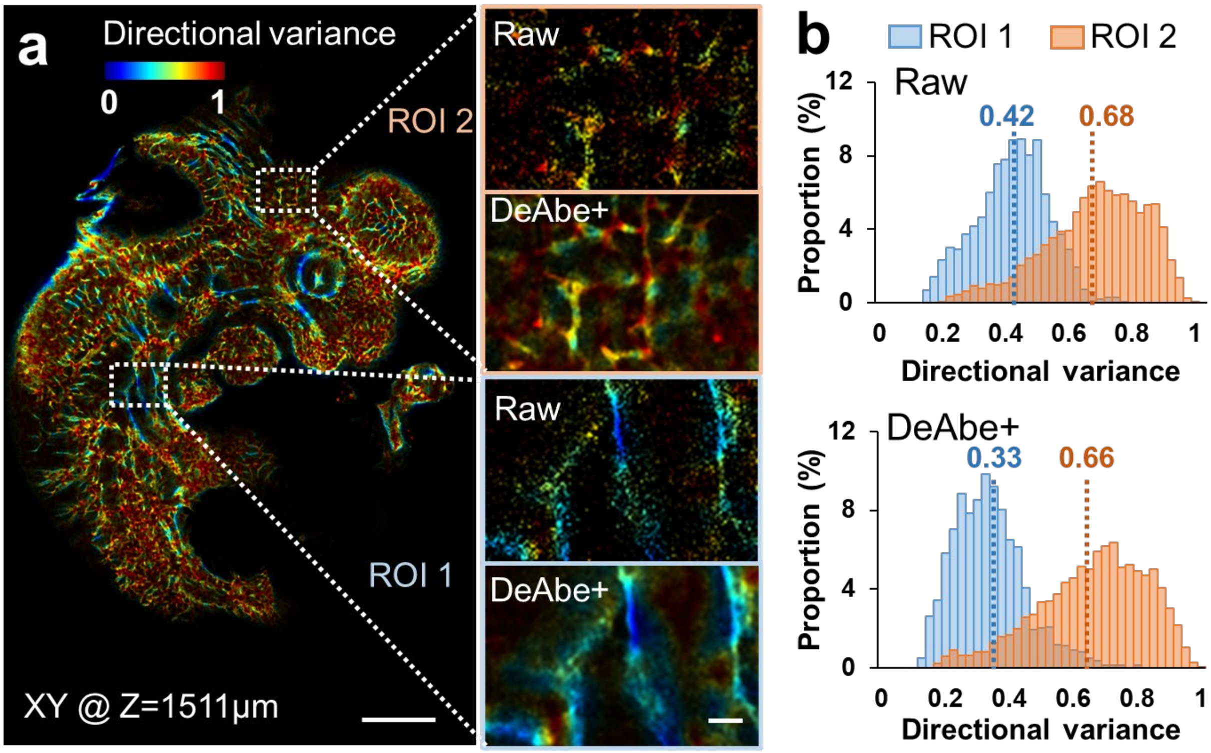

Supplementary Fig. 12, Comparing directional variance between raw and DeAbe+ restoration. a) Data are as in **Fig. 3g**, but higher magnification views of each ROI also show directional variance computed from raw data in addition to DeAbe+. Note noisier appearance in raw data, which results in less separation between histograms (**b**). Scale bars: 300 μm **a)**, 50 μm inset.

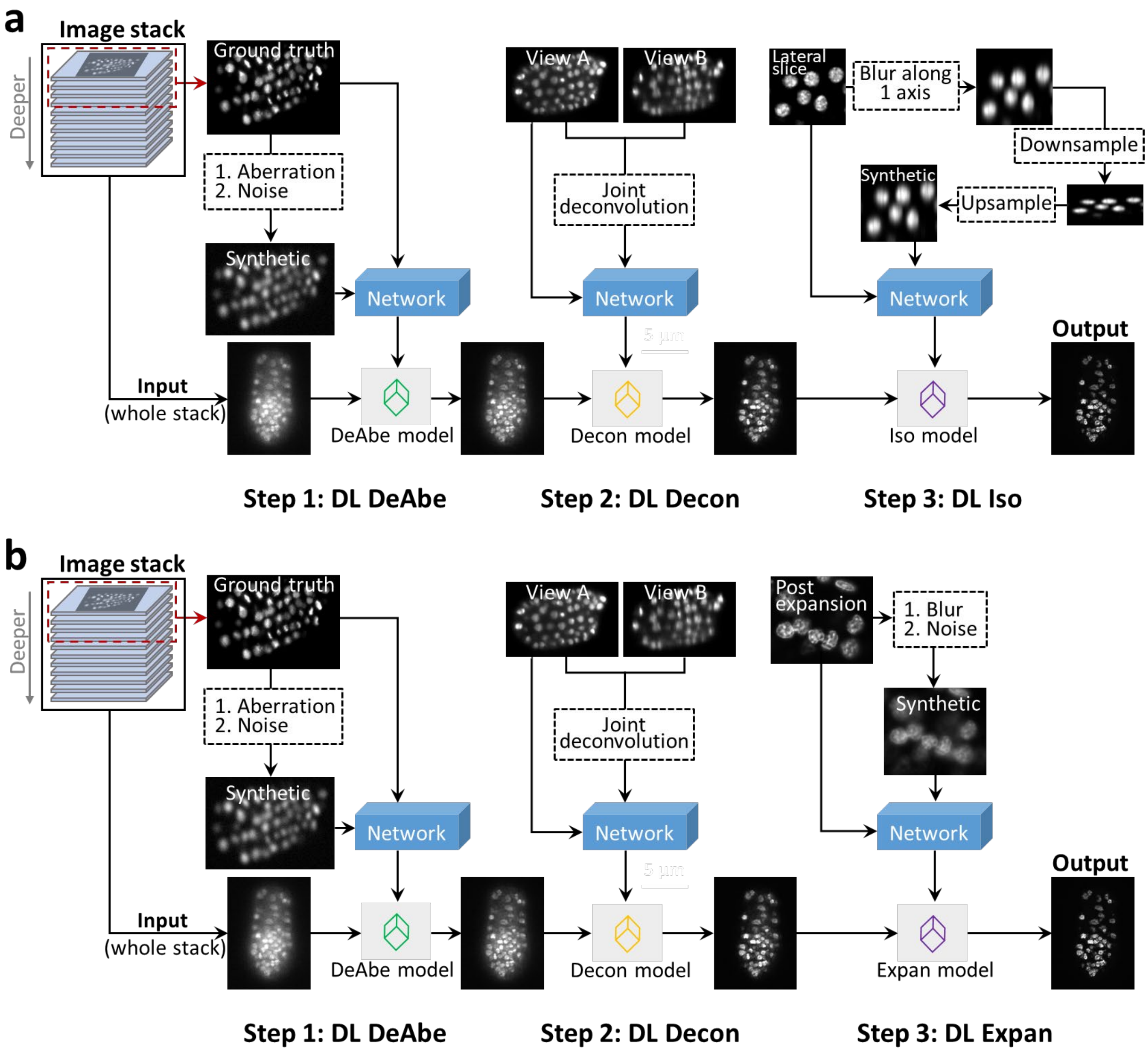

Supplementary Fig. 13, Multi-step image restoration schemata. We trained independent neural networks to compensate for aberrations (Step 1: DL DeAbe, see also **Fig. 1a**), deconvolve the data (Step 2: DL Decon), and to super-resolve the data (Step 3: DL Iso, **a)** or DL Expan, **b)**). For the deconvolution networks, we used high quality joint deconvolution based on orthogonal views (after DL DeAbe processing) from diSPIM as ground truth and trained the network to predict this joint deconvolution given only single-view input. For the isotropic enhancement model in **a)**, we synthetically blur lateral views to resemble axial views and downsample and upsample the data to mimic the coarser pixel size in z. We then train a neural network to reverse this degradation. For the expansion microscopy network in **b)**, we synthetically degraded high-resolution images from expanded samples until the synthetic data resembled conventional images acquired on the diSPIM and trained a network to reverse this degradation. Serial application of each network produced the final prediction. See also **Methods**.

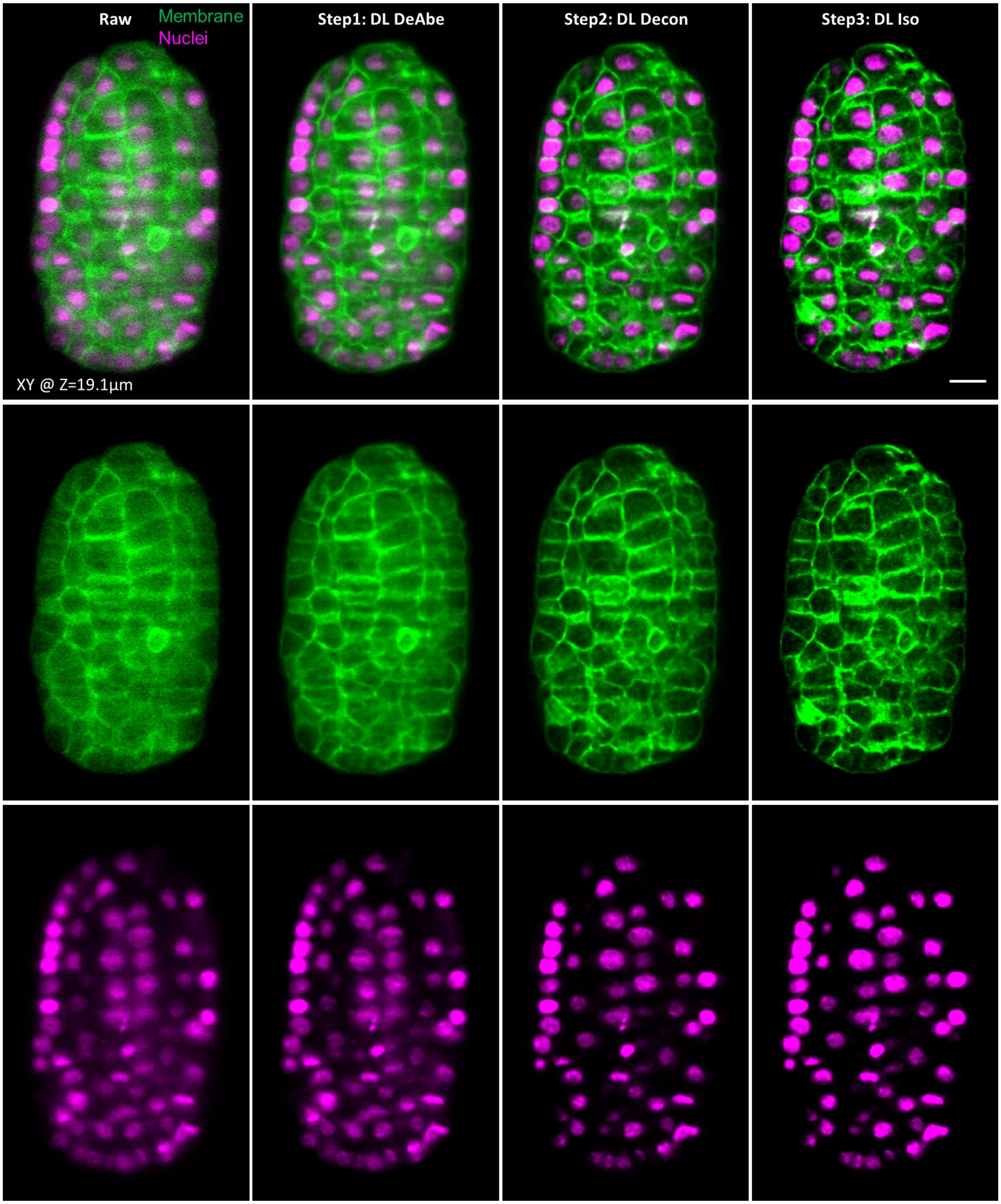

Supplementary Fig. 14, Improvements in lateral views after multi-step deep learning. Data from *C. elegans* embryos expressing membrane and nuclear markers as in **Fig. 4a**, emphasizing improvements to lateral views (single plane 19.1 µm into the volume) corresponding to successive steps in restoration

(columns). Merged views (top), membrane channel (middle), and nuclear channel (bottom) are shown. Scale bar: 5 μm.

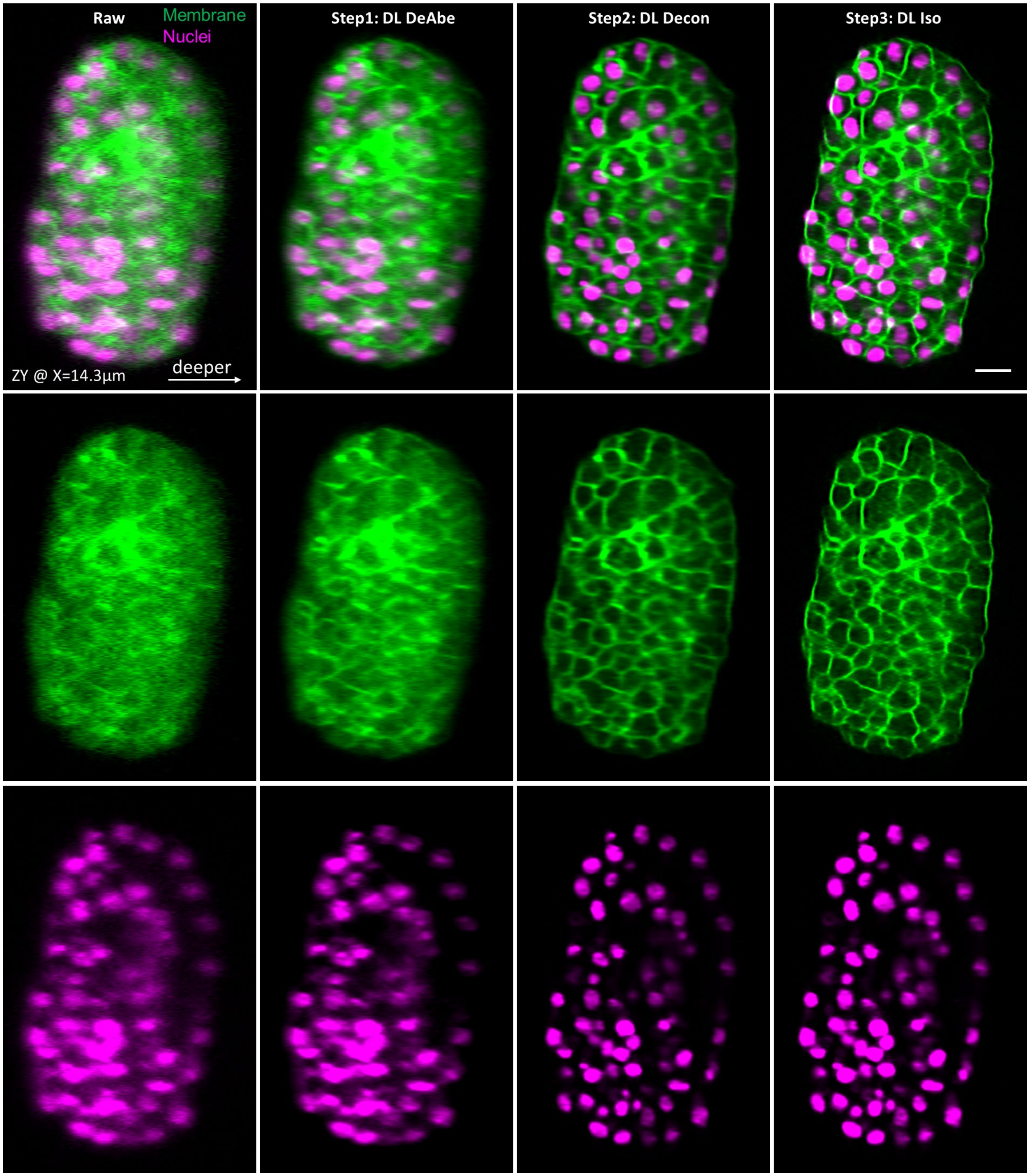

Supplementary Fig. 15, Improvements in axial views after multi-step deep learning. Data from *C. elegans* embryos expressing membrane and nuclear markers as **Fig. 4a**, emphasizing improvements to axial views (single plane 14.3 μm into the volume) corresponding to successive steps in restoration (columns). Merged views (top), membrane channel (middle), and nuclear channel (bottom) are shown. Scale bar: 5 μm.

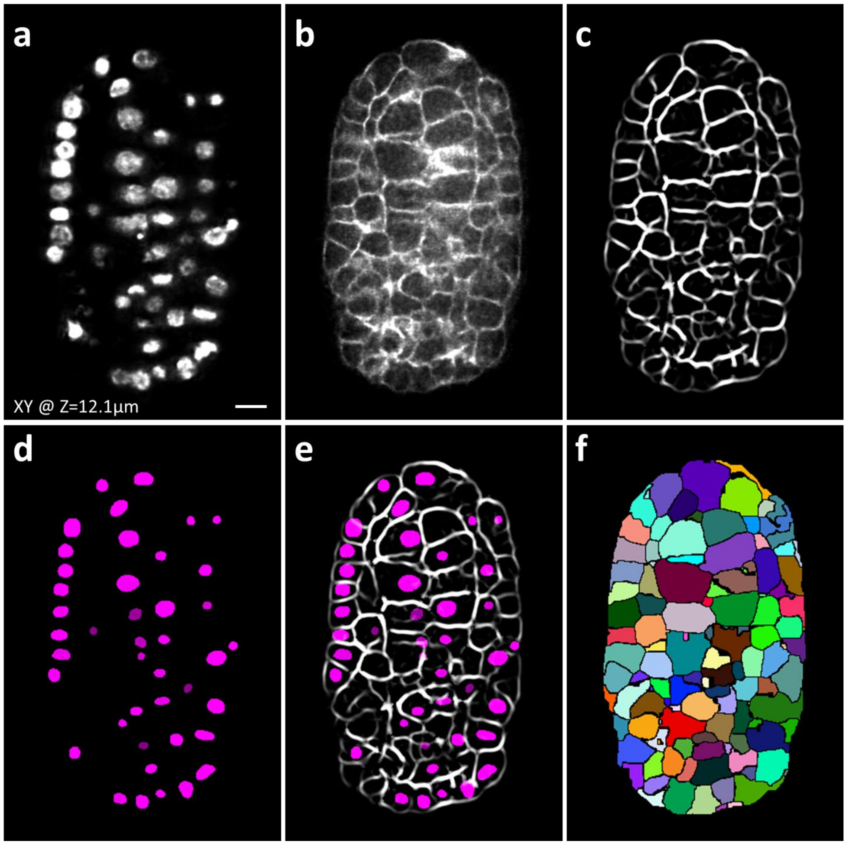

Supplementary Fig. 16, Automated membrane segmentations on restored image volumes derived from dual-color (pan-nuclear and membrane) *C. elegans* embryos. a, b) Image planes from nuclear and membrane channels from representative volume as in **Fig. 4a-d**, after multistep image restoration. **c)** Membrane-enhanced image after applying vascular structure enhancement filter to **b**. **d)** Segmented nuclei with Mask RCNN. **e)** Overlay of enhanced membrane and segmented nuclei. **f)** The associated multi-instance segmentation of cell boundaries. Scale bar: 5 μm.

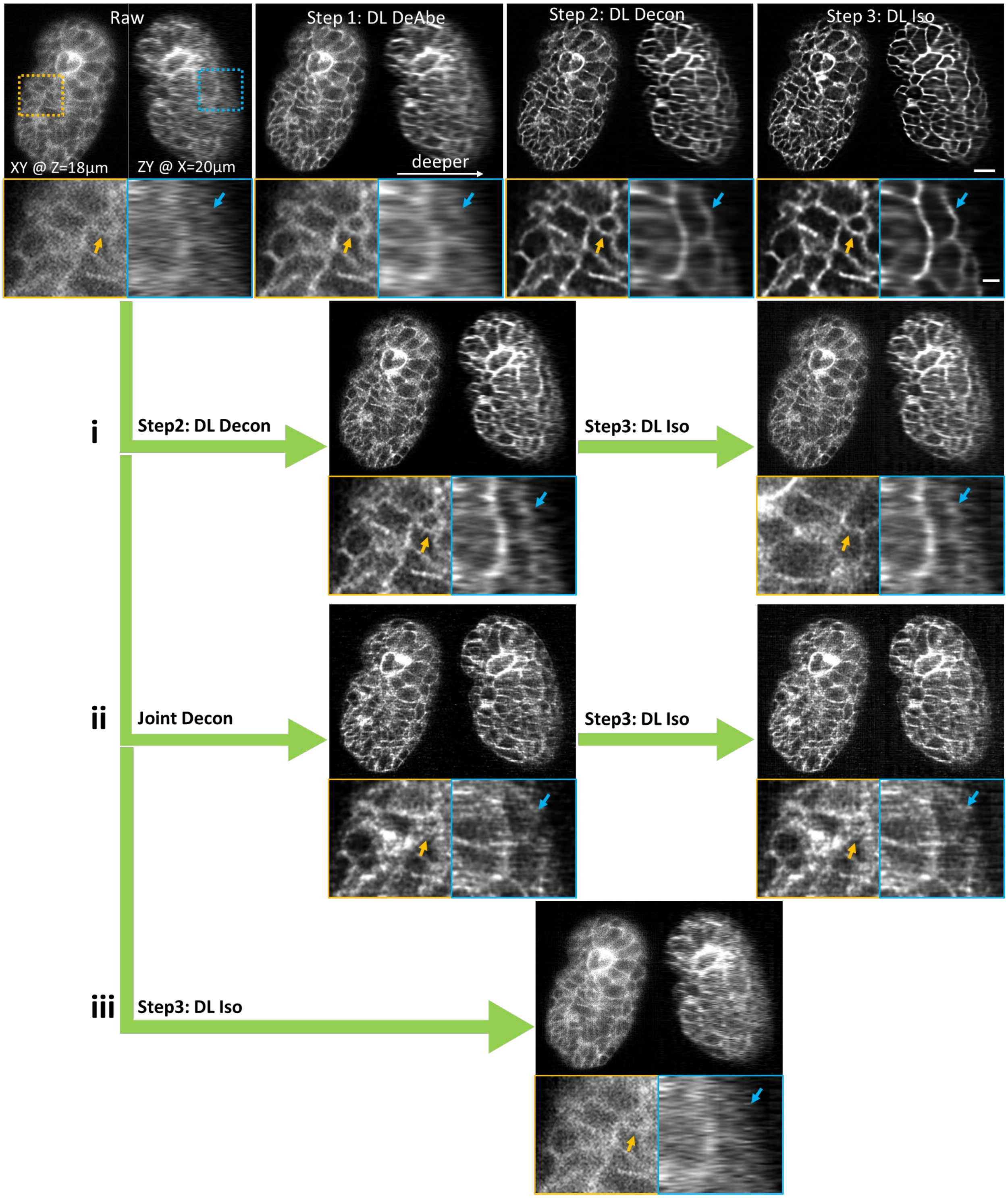

Supplementary Fig. 17, Ablation experiments highlighting value of DeAbe model in multi-step restoration. Top rows show data from membrane channel of *C. elegans* embryos as in **Fig. 4a**, highlighting progressive improvements in image quality after each step of multi-step restoration, including higher magnification insets in second row corresponding to dashed rectangular regions in top row. Data are also shown (i) if DeAbe (Step 1) is ablated, proceeding directly from raw data to Steps 2

and 3; (ii) if DeAbe is ablated, and joint deconvolution used in lieu of the DL Decon (Step 2); (iii) if steps 1 and 2 are both ablated, and raw data is used directly as in the input for the DL Iso network (Step 3). The final prediction is degraded in schemes (i) – (iii) relative to the original method, as membranes are either noisy or not well resolved (arrows). Scale bars: 5 μm, 2 μm inset.

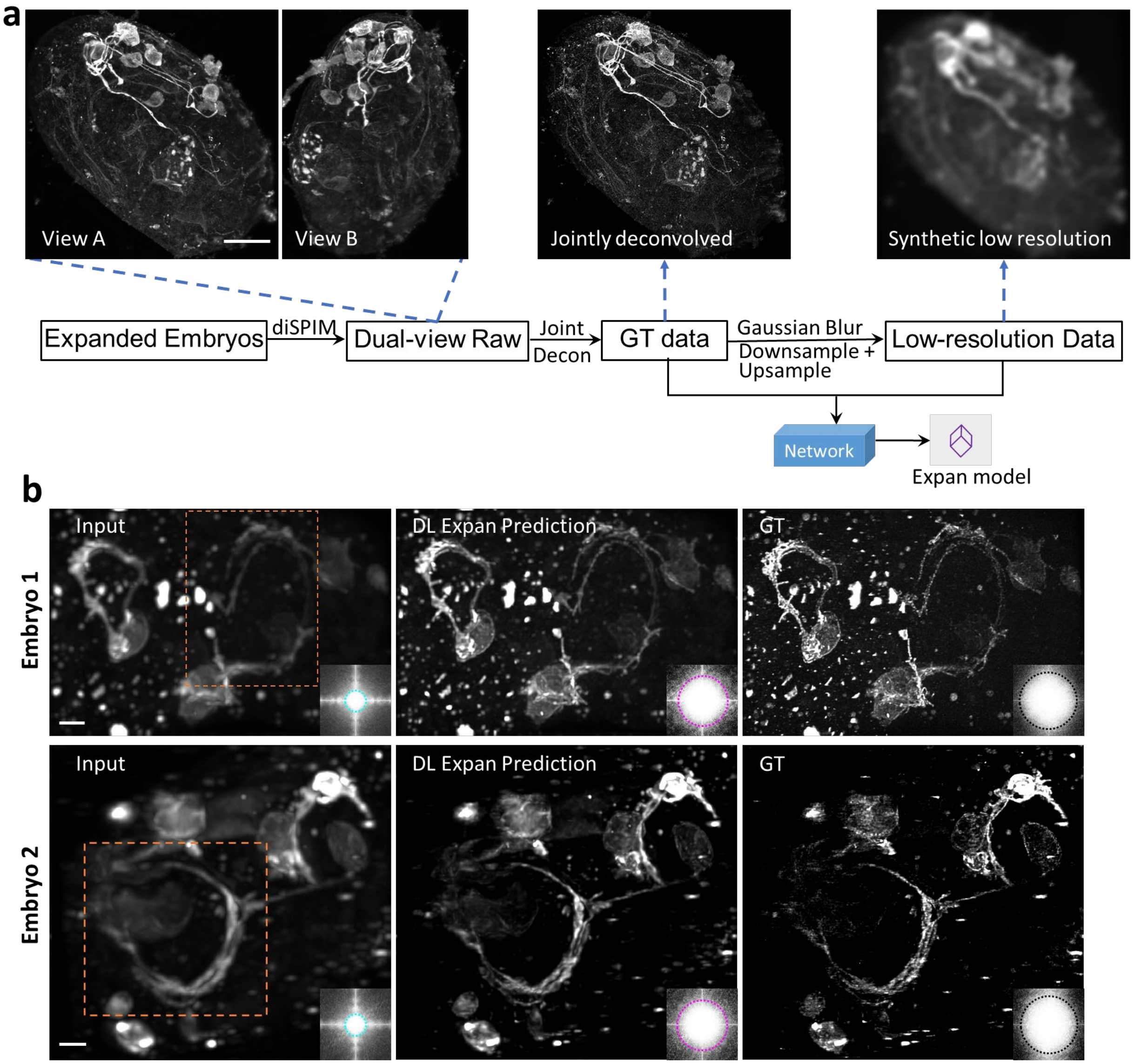

Supplementary Fig. 18, Using expanded embryos as high resolution ground truth for training resolution enhancement model. a) Expansion was used to enlarge embryos expressing ttx-3B-GFP 3.6-fold and the samples were then imaged using diSPIM. The two views (A and B) were registered, and joint deconvolution applied to produce volumes with more nearly isotropic resolution. These volumes were synthetically degraded via blurring and downsampling to produce semisynthetic data with resolution more like the raw data prior to expansion, and used to train a network (Expan model) for resolution enhancement. In these images, GFP signal was boosted using immunolabeling. **b)** Example images from two embryos highlighting nerve ring region (orange dashed rectangles), comparing synthetic, low-resolution input data (left), prediction from DL Expan model, and ground truth data. The Fourier transform images are shown in the insets, with cyan, pink, and black dotted circles corresponding to the spatial resolutions of 330 nm, 130 nm, and 105 nm respectively. Scale bars: 2 μm.

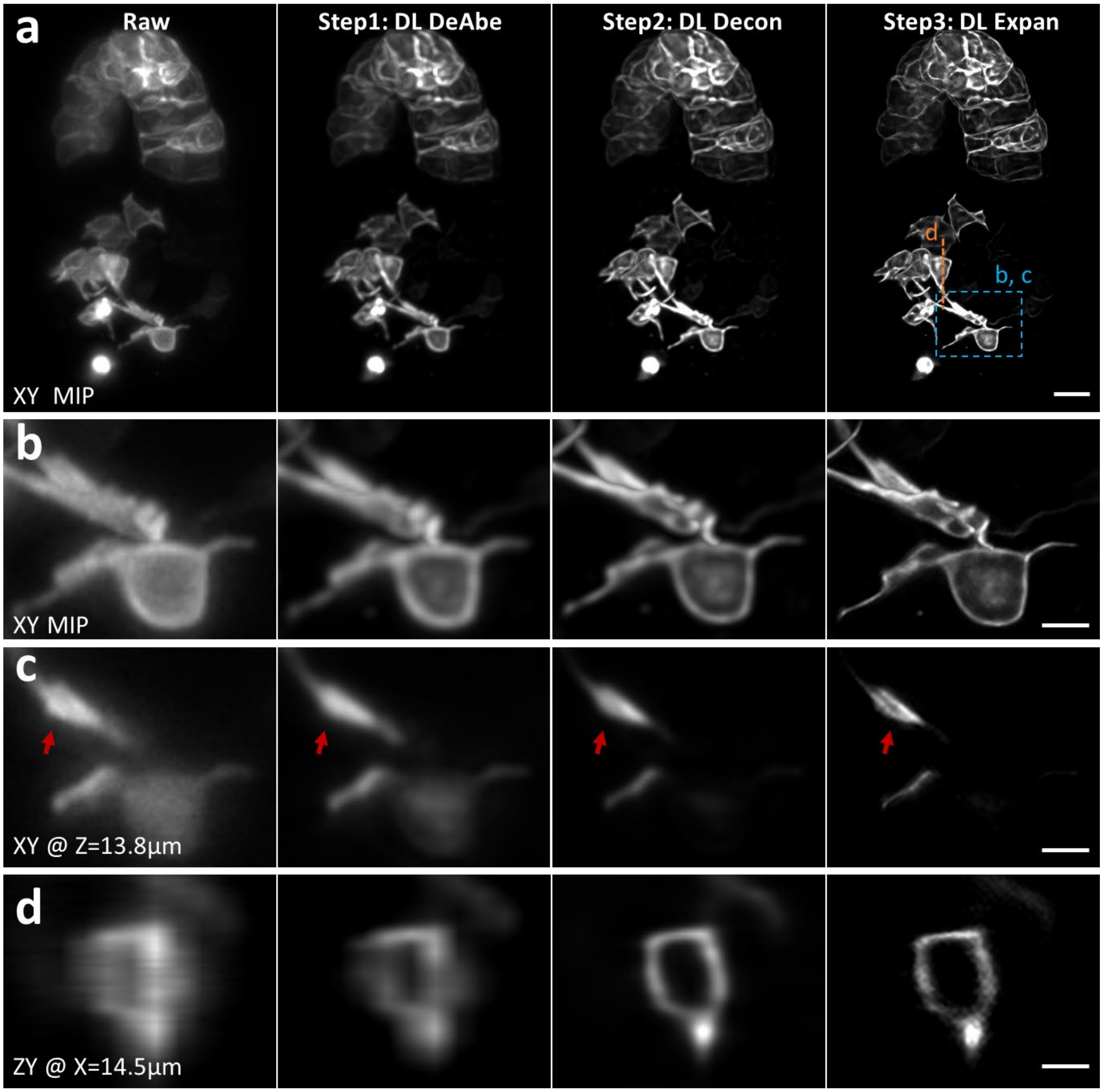

Supplementary Fig. 19, Progressive improvements in image quality in multi-step image restoration. a) *C. elegans* embryos expressing membrane markers in neurons and gut cells were imaged as in **Fig. 4e** and passed through our multi-step image restoration pipeline. Higher magnification lateral **b, c)** and axial **d)** views of dashed blue rectangle and orange line in **a)** are also shown. Red arrows highlight features resolved after the third step. MIP: maximum intensity projection. Scale bars: 500 µm **a)**; 100 µm **b, c, d)**.

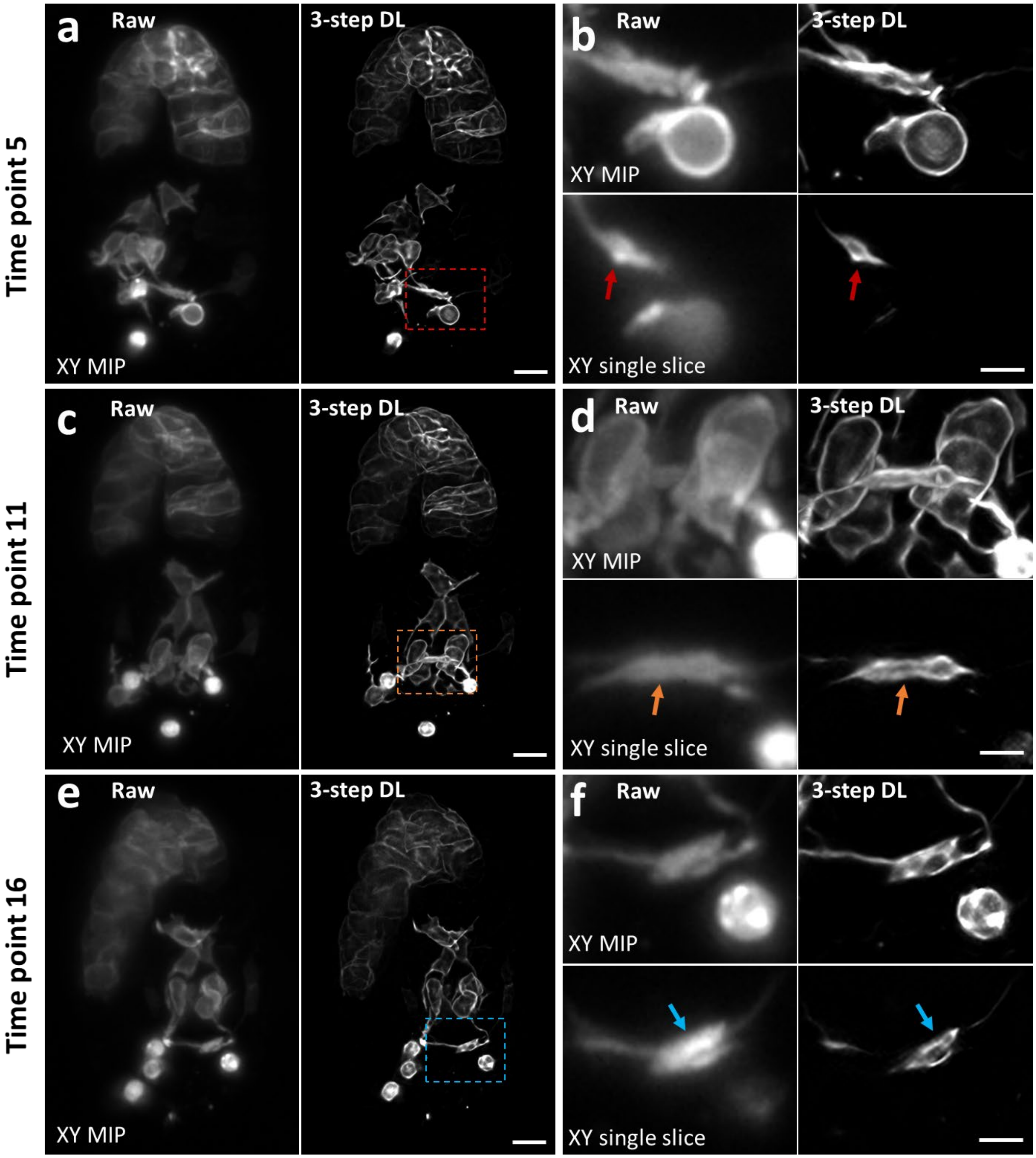

Supplementary Fig. 20, Additional examples of improved image quality after multi-step image restoration. **a)** Selected time points from the dataset highlighted in **Fig. 4e-i**, further comparing raw data (left) and multi-step image restoration (3-step DL, right). Higher magnification views of dashed red **b)**, orange **c)**, and blue **d)** rectangles in **a)** are also shown in lateral maximum intensity projections (top) or single planes (bottom). Arrows indicate features that are obscured in raw data but better resolved after image restoration. Scale bars: 500 μm **a, c, e)**; 100 μm **b, d, f)**.

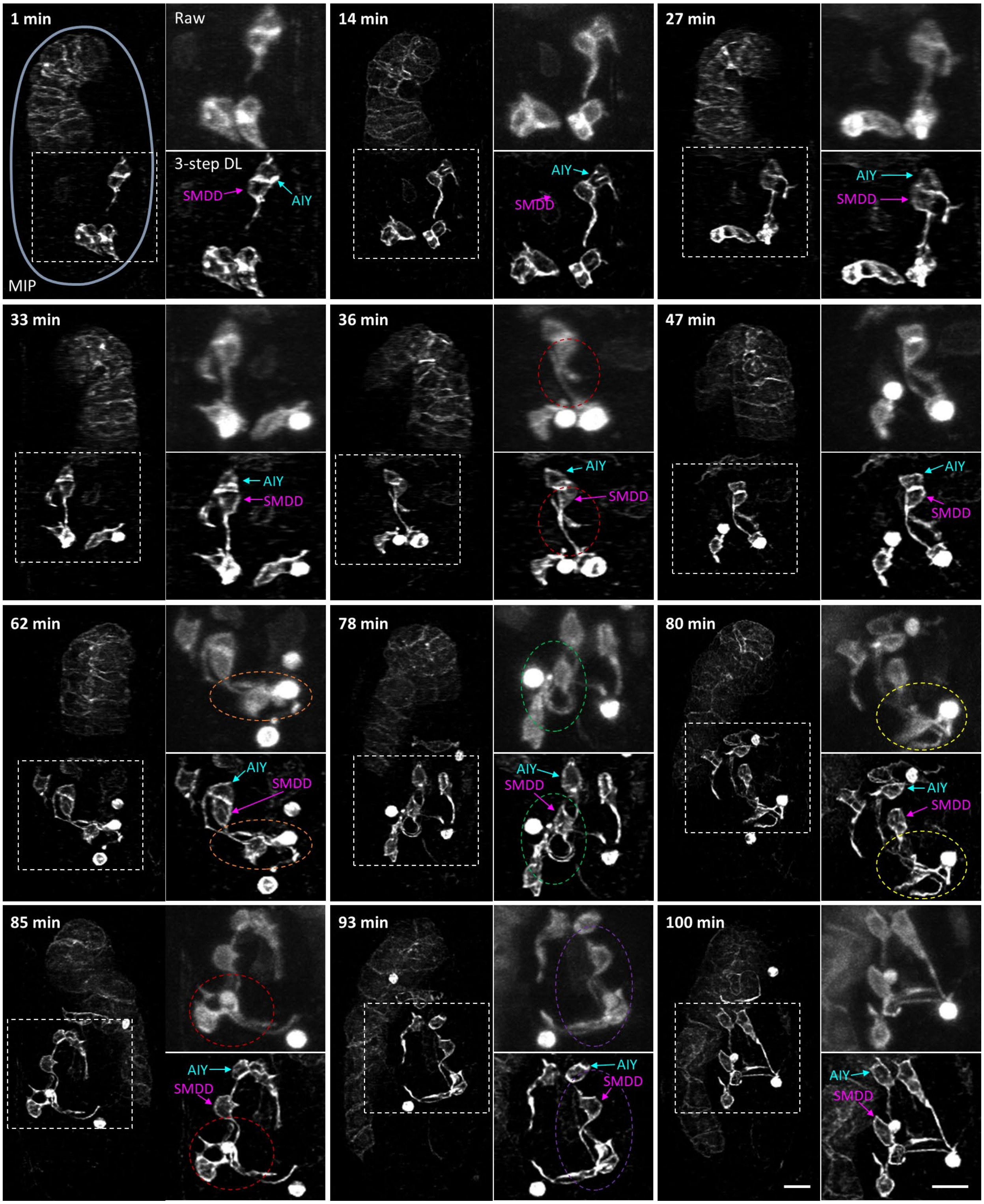

Supplementary Fig. 21, Visual inspection of AIY and SMDD neurites is facilitated after multi-step image restoration. Example images in support of **Fig. 4i** at indicated time points, highlighting additional clarity after image restoration. Higher magnification views of dashed white rectangles are shown at

right, comparing raw (upper) and restored (lower) data. AIY and SMDD cell bodies have been indicated with arrows for clarity; dashed circles highlight associated neurites as they innervate the nerve ring area. Scale bars: 5 μm.

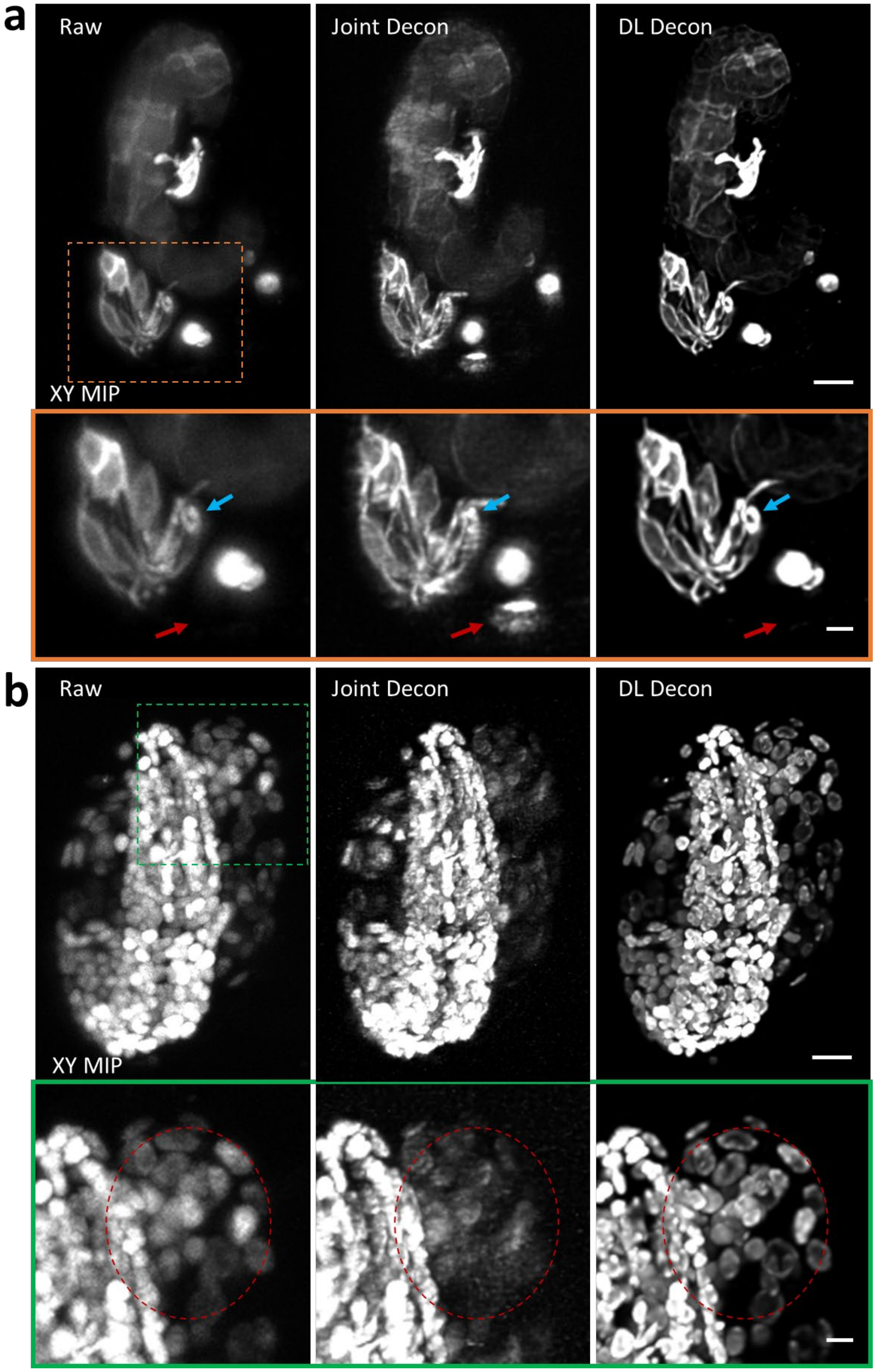

Supplementary Fig. 22, Multi-step image restoration does not suffer motion artifacts like multiview fusion. a) Maximum intensity projection (MIP) images of *C. elegans* embryos expressing ttx-3B-GFP, imaged with diSPIM, comparing raw single-view recordings (Raw, left), dual-view fusion result via traditional joint deconvolution (Joint Decon, middle) and deep learning-based deconvolution after

application of DeAbe (DL Decon, right). Higher magnification lateral views of orange dotted rectangular region are shown in the bottom panel, with cyan and red arrows highlighting motion induced artifacts present in Joint Decon images but not Raw or DL Decon images. **b**) Images of *C. elegans* embryos expressing pan-nuclear GFP marker, imaged with symmetric diSPIM. Higher magnification lateral views of green dotted rectangular region are shown in the bottom panel, with red dotted circles highlighting the motion induced artifacts present in Joint Decon images but not Raw or DL Decon images. Scale bars: 5 μm, 2 μm insets.

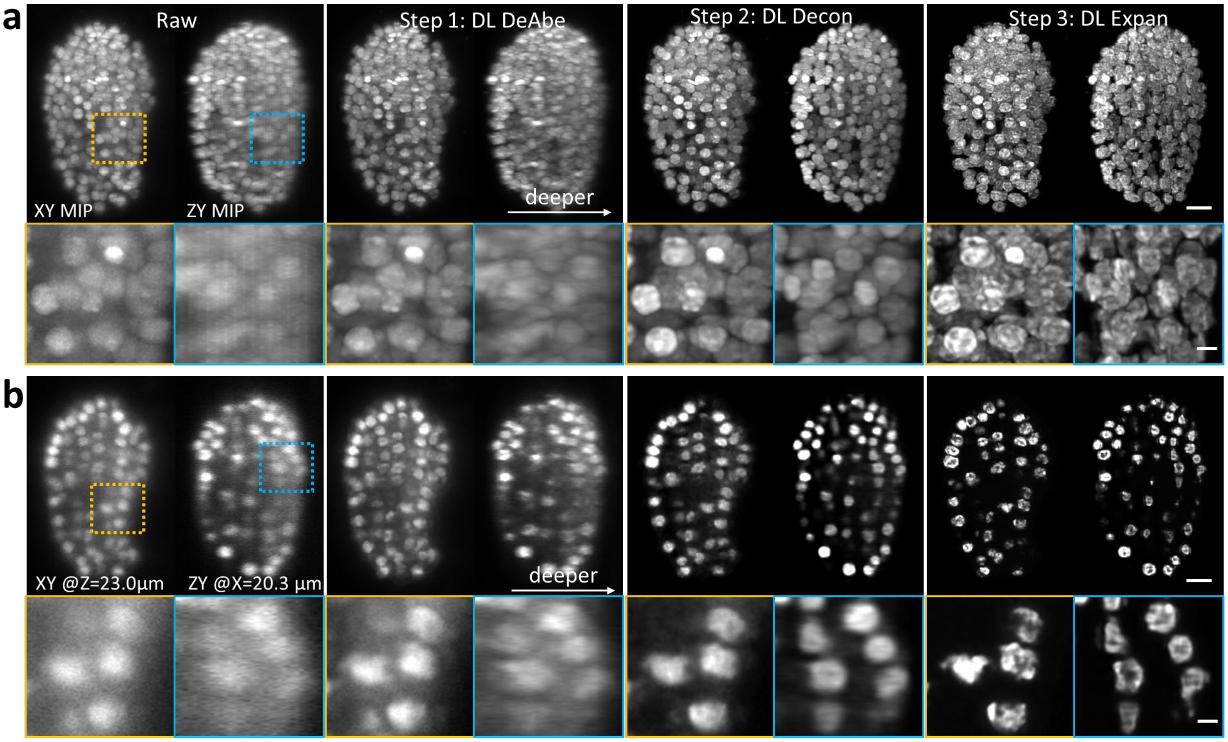

Supplementary Fig. 23, Additional example of multi-step restoration. a) *C. elegans* embryos expressing pan-nuclear GFP marker were imaged with high numerical aperture diSPIM and input into our multi-step imaging restoration pipeline. Top row: Comparing raw, after DeAbe (Step 1), after additionally applying deconvolution network (Step 2), and finally after additionally applying DL Expan model (Step 3). See also **Methods** for further details on sample preparation and imaging system. Higher magnification views of lateral (left, corresponding to dashed orange rectangle in top row) and axial (right, corresponding to dashed blue rectangle in top row) maximum intensity projections (MIP) are also shown. **b)** As in **a)**, but highlighting single lateral and axial imaging planes instead of MIPs. Scale bars: 5 μm, 2 μm insets. See also **Supplementary Videos 13, 14.**

Supplementary Table 1, Sample information and parameters used in generating DeAbe models.

Samples	Synthetic phantoms	NK-92 cells	*C. elegans* embryos					Adult *C. elegans*[3]	Live mouse cardiac tissue	Fixed mouse liver tissue	Cleared mouse embryo[3]
**Figures/Videos**	Fig 1b, Supp Figs 1-5 & Supp Videos 1,2	Fig 2b-d, Supp Fig 8 & Supp Video 5	Fig 2a & Supp Video 3	Fig. 4e-i, Supp Figs 19-21, 22a & Supp Video 12	Supp Fig 22b & Supp Videos 13,14	Supp Figs 6, 17	Fig. 4a-d, Supp Figs 14-16[3]	Supp Fig 7 & Supp Video 4	Fig. 2e, f, Supp Fig 9 & Supp Video 6	Supp Video 7	Fig. 3, Supp Figs 10-12 & Supp Videos 8, 9
**Label**	--	Wheat germ agglutinin (WGA)	Nuclei	Neurites	Nuclei	Membranes	Membranes, Nuclei	Neurons, 4 color channels	Mitochondria	Membranes	Vessels, neurons
**Microscopy**	Light sheet microscopy	iSIM	iSPIM	iSPIM	iSPIM	iSPIM	iSPIM	Spinning disk confocal microscopy	Two-photon microscopy	Two-photon microscopy	Confocal microscopy
**Detection NA**	1.1	1.3	1.1	0.8	0.8	0.8	1.1	1.2	1.0	1.0	0.5
**Maximum depth of raw volumes**	33 μm	50 μm	33 μm	33 μm	33 μm	33 μm	33 μm	32 μm	200 μm	72 μm	~2200 μm
**Depth of shallow subvolumes**	33 μm	~25 μm	12 μm	12 μm	12 μm	12 μm	15 μm	18 μm	35 μm	~30 μm	~1000 μm
**Size of shallow subvolumes**	256x256x256 voxels x 50 subvolumes	~542x540x68 voxels x 18 subvolumes	~324x410x20 voxels x 465 subvolumes	~256x384x16 voxels x 623 subvolumes	~248x354x14 voxels x 386 subvolumes	~290x364x16 voxels x 622 subvolumes	~256x470x32 voxels x 404 subvolumes	512x256x12 voxels X 153 subvolumes	1024x1024x64 voxels x 24 subvolumes	~1024x1024x50 voxels x 24 subvolumes	~740x620x180 voxels x 26 subvolumes (From 4 embryos)
**Training data size[1] (32 bit/voxel)**	62 GB	27 GB	88 GB	73.4 GB	28 GB	87 GB	145 GB	18 GB	120 GB	95 GB	99 GB
**Training time cost**	27 h	18 h	26 h	20 h	14 h	16 h	37 h	10 h	44 h	39 h	59 h
**Applying volume size[2]**	256x256x256 voxels	367x376x200 voxels	340x460x59 voxels	~256x384x50 voxels	~208x311x41 voxels	~270x370x45 voxels	340x510x70 voxels	512x256x21 voxels	1024x1024x304 voxels	1024x1024x140 voxels	1440x1740x433 voxels
**Applying time cost**	39 s	31 s	23 s	7	3 s	7	23 s	11 s	5.7 min	2.5 min	15 min

All samples and datasets used for training the DeAbe models in this paper. The time costs are reported based on tests on a Windows 10 workstation (CPU: Intel Xeon, Platinum 8369B, two processors; RAM: 256 GB; GPU: NVIDIA GeForce RTX 3090 with 24 GB memory). With additional footnotes:

1: The training data consists of paired GT images and synthetically aberrated images.

2: Representative single volume for testing the applying time cost.

3: For multicolor images, data size and time cost are reported for single color channel.

Supplementary Note 1, Synthetic generation of aberrated data from fluorescence images

Basic concept

The key insight of our approach is that fluorescence images acquired on the 'near side' of three-dimensional volumes are often close to diffraction limited. Such images thus represent ground truth data that can be used to train neural networks to reverse the effect of synthetically introduced aberrations. For this purpose, we intentionally synthetically aberrate the images acquired by fluorescence microscopes with a 'forward model'.

We assume a scalar imaging model with pupil function $H(r,\theta)$ given by

$$H(r,\theta) = |P(r,\theta)|e^{i\phi(r,\theta)} \tag{1}$$

$$r = \sqrt{u^2 + v^2}, \tag{2}$$

where u, v are pupil plane coordinates, r and θ are the radial and angular coordinates in the pupil plane, $\phi(r,\theta)$ is the phase aberration introduced either in the data or synthetically, and $P(r,\theta)$ is a binary pupil mask that spans the set of spatial frequencies transmitted by the imaging system:

$$P(r,\theta) = \begin{cases} 1, \; for\; r \leq \dfrac{NA}{\lambda} \\ 0, otherwise\,, \end{cases} \tag{3}$$

where NA is the objective numerical aperture and λ is the imaging wavelength.

The phase aberration, or wavefront distortion, can be conveniently expressed using Zernike basis functions $\phi_m(r,\theta)$ and associated coefficients c_m :

$$\phi(r,\theta) = \sum_{m=0}^{M} c_m \phi_m(r,\theta)\,. \tag{4}$$

The corresponding Root Mean Square (RMS) wavefront distortion is calculated as

$$RMS = \sqrt{\frac{1}{\int P(r,\theta)drd\theta}\int \left[\phi(r,\theta) - \overline{\phi(r,\theta)}\right]^2 drd\theta} = \sqrt{\sum_{m=3}^{M} {c_m}^2}\,, \tag{5}$$

where $\overline{\phi(r,\theta)}$ is the mean wavefront, and the first three terms representing piston (m=0), tip (m=1) and tilt (m=2) are ignored.

The three-dimensional coherent point-spread function associated with this pupil function is given by[1]

$$h(x,y,z) = \mathcal{F}^{-1}\{H(r,\theta)e^{iz\gamma(r)}\}, \tag{6}$$

$$\gamma(r) = \frac{2\pi n}{\lambda}\sqrt{1 - \left(\frac{\lambda r}{n}\right)^2}\,, \tag{7}$$

where x, y denote lateral coordinates, z the axial coordinate and n the refractive index. The incoherent PSF (appropriate for widefield fluorescence microscopy) is then given by

$$s(x,y,z) = |h(x,y,z)|^2. \tag{8}$$

Given an object $f(x,y,z)$ we can then describe the forward imaging model as:

$$d(x,y,z) = s(x,y,z) \otimes f(x,y,z), \tag{9}$$

where ⊗ is the convolution operation.

Omitting the (x,y,z) coordinates for simplicity, the forward imaging model

$$d = s \otimes f \tag{10}$$

can be Fourier transformed ($\mathcal{F}(\quad)$) to obtain

$$\mathcal{F}(d) = \mathcal{F}(s)\mathcal{F}(f), \tag{11}$$

where the Fourier transform of the PSF, $\mathcal{F}(s)$ is also known as the optical transfer function (OTF). If $\phi(r)$ = 0, i.e., the wavefront is 'flat' or unaberrated, we obtain the diffraction-limited image d_0 from the ideal PSF s_0 and object f as

$$\mathcal{F}(d_0) = \mathcal{F}(s_0)\mathcal{F}(f). \tag{12}$$

Eliminating the object term using the last two equations,

$$\mathcal{F}(d) = \mathcal{F}(d_0)\left[\frac{\mathcal{F}(s)}{\mathcal{F}(s_0)}\right]. \tag{13}$$

To prevent division by zero, we modify the denominator by adding a small value α ($\alpha = 0.01$ for all datasets in this paper). Inverse Fourier transforming and optionally adding a noise term we obtain finally

$$d = \mathcal{F}^{-1}\left\{\mathcal{F}(d_0)\left[\frac{\mathcal{F}(s)}{\mathcal{F}(s_0) + \alpha}\right]\right\} + noise. \tag{14}$$

This equation provides a prescription for deriving an aberrated image d from a diffraction-limited image d_0 and the ratio of aberrated OTF $\mathcal{F}(s)$ to diffraction limited OTF $\mathcal{F}(s_0)$. The degree of aberration can be tuned by adjusting the Zernike functions $\phi_m(r)$ and magnitude of their associated coefficients c_m.

In practice, we use images from the near side of fluorescence microscopy volumes as d_0 and tune c_m until we obtain d that resemble aberrated images that occur on the 'far side' of the image stack (see **Supplementary Table 1** for relevant parameters used in this paper).

Extension to different microscopes

To adopt this concept for other microscopes, we modify the PSF in equation (8), extending the wide-field PSF as needed to more accurately model light-sheet microscopy, confocal microscopy, instant SIM, and two-photon microscopy. Correspondingly, we re-write the equation (8) by adding a subscript "WF" to explicitly indicate it as using the wide-field PSF:

$$s_{WF} = s_{WF}(x,y,z) = |h(x,y,z)|^2. \tag{15}$$

We leave $s(x,y,z)$ as the generalized system PSF, which is constructed by considering the excitation PSF and emission PSF:

$$s = PSF_{sys} = PSF_{exc} \times PSF_{em}. \tag{16}$$

By substituting equation (16) into equation (10), we obtain the forward model of each microscope.

*1) Light sheet micro*scopy

In light sheet microscopy, the emission PSF is equivalent to the wide-field PSF and the excitation PSF is often (as in these experiments) modeled as a virtual sheet constructed by scanning a low numerical aperture Gaussian beam across the field of view. We model the excitation sheet as uniform in the lateral directions but Gaussian in the axial direction (lateral and axial are defined from the perspective of the detection objective):

$$PSF_{exc} = \frac{1}{\sigma\sqrt{2\pi}} e^{-\frac{z^2}{2\sigma^2}}. \tag{17}$$

Then the final system PSF is

$$s_{LS} = PSF_{exc} \times PSF_{em} = \frac{1}{\sigma\sqrt{2\pi}} e^{-\frac{z^2}{2\sigma^2}} \times s_{WF} = A s_{WF} e^{-\frac{z^2}{2\sigma^2}}, \tag{18}$$

where A is a constant which is discarded as we normalize the system PSF by integrating its intensity to 1. In practice, by measuring the thickness of the light sheet, i.e., the full width at half maximum (FWHM) in the axial direction at the beam waist, σ can be estimated based on the assumption that the beam is Gaussian as:

$$\sigma = \frac{FWHM_{LS}}{2\sqrt{2ln2}} = \frac{FWHM_{LS}}{2.3548}. \tag{19}$$

2) Confocal microscopy

We construct the confocal PSF as:

$$s_{confocal} = PSF_{exc} \times PSF_{em} = s_{WF(\lambda_1)} \times \left(s_{WF(\lambda_2)} \otimes s_{Pinhole}\right), \tag{20}$$

where $s_{WF(\lambda 1)}$ and $s_{WF(\lambda 2)}$ are wide-field PSFs with excitation wavelength λ_1 and emission wavelength λ_2, respectively and $\otimes$ is the convolution function. $s_{Pinhole}$ models the physical pinhole in a confocal system as a binary circular mask at the z=0 plane:

$$s_{Pinhole}(x, y, z) = \begin{cases} 1, \; for\ z = 0\ and\ \sqrt{x^2 + y^2} \le Pinhole\ size \\ 0, \;\; otherwise. \end{cases} . \tag{21}$$

In practice, we implement the convolution operation in equation (20) in the Fourier domain, giving:

$$s_{confocal} = s_{WF(\lambda_1)} \times \mathcal{F}^{-1}\left\{\mathcal{F}\left(s_{WF(\lambda_2)}\right) \times \mathcal{F}\left(s_{Pinhole}\right)\right\}. \tag{22}$$

Note that we do not model the multifocal and pinhole lattice in spinning-disk confocal microscopy, so the model used for this form of microscopy is the same as what is used in point-scanning confocal microscopy.

3) Instant SIM

To simplify calculations, we neglected to model the multifocal illumination and pinhole lattice, instead modeling the PSF as a confocal PSF with infinitely small pinhole (a Dirac Delta function). In this case, the final super-resolution PSF is approximated by:

$$s_{iSIM} = \left(s_{WF(\lambda_1)} \otimes s_{Pinhole}\right) \times s_{WF(\lambda_2)} = s_{WF(\lambda_1)} \times s_{WF(\lambda_2)}. \quad (23)$$

Again, $s_{WF(\lambda 1)}$ and $s_{WF(\lambda 2)}$ are the wide-field PSFs with excitation wavelength λ_1 and emission wavelength λ_2, respectively.

4) Two-photon microscopy

For two-photon microscopy, the excitation PSF is the square of the wide-field PSF with the excitation wavelength. The emission PSF is treated as uniform as there is typically no confinement or modulation on the emission side, and this constant can be discarded as the final system PSF is normalized as described above. Therefore, we have:

$$s_{2P} = PSF_{exc} \times PSF_{em} = PSF_{exc} = {s_{WF(\lambda_1)}}^2. \quad (24)$$

For most datasets employing single photon microscopy, we used 488 nm excitation and 532 nm excitation; for two-photon microscopy data, we used 960 nm wavelength excitation.

1 Hanser, B. M., Gustafsson, M. G. L., Agard, D. A. & Sedat, J. W. Phase retrieval for high-numerical-aperture optical systems. *Optics Letters* **28**, 801-803 (2003).
